# Cannabinoids Reduce Extracellular Vesicle Release from HIV-1 Infected Myeloid Cells and Inhibit Viral Transcription

**DOI:** 10.3390/cells11040723

**Published:** 2022-02-18

**Authors:** Catherine DeMarino, Maria Cowen, Pooja Khatkar, Bianca Cotto, Heather Branscome, Yuriy Kim, Sarah Al Sharif, Emmanuel T. Agbottah, Weidong Zhou, Cecilia T. Costiniuk, Mohammad-Ali Jenabian, Cohava Gelber, Lance A. Liotta, Dianne Langford, Fatah Kashanchi

**Affiliations:** 1 Laboratory of Molecular Virology, School of Systems Biology, George Mason University, Manassas, VA 22030, USA; catherine.demarino@nih.gov (C.D.); maria.cowen@nih.gov (M.C.); pkhatkar@gmu.edu (P.K.); hbransco@gmu.edu (H.B.); ykim78@gmu.edu (Y.K.); 2 Department of Neuroscience, Lewis Katz School of Medicine at Temple University, Philadelphia, PA 19140, USA; bcotto@mail.rockefeller.edu (B.C.); tdl@temple.edu (D.L.); 3 Department of Clinical Laboratory Sciences, College of Applied Medical Sciences, King Saud Bin Abdulaziz, University for Health Sciences, Jeddah 22384, Saudi Arabia; salshar@gmu.edu; 4 Montgomery College, Takoma Park, MD 20912, USA; emmanuel.agbottah@montgomerycollege.edu; 5 Center for Applied Proteomics and Molecular Medicine, George Mason University, Manassas, VA 20110, USA; wzhou@gmu.edu (W.Z.); liotta.lance@gmail.com (L.A.L.); 6 Infectious Diseases and Immunity in Global Health Program, Research Institute of McGill University Health Centre, Montreal, QC H4A 3J1, Canada; cecilia.costiniuk@mcgill.ca; 7 Department of Microbiology and Immunology, McGill University, Montreal, QC H3A 2B4, Canada; 8 Department of Biological Sciences and CERMO-FC Research Centre, Université du Québec à Montréal, Montreal, QC H3C 3J7, Canada; jenabian.mohammad-ali@uqam.ca; 9 Serpin Pharma, Manassas, VA 20109, USA; cgelber@serpinpharma.com

**Keywords:** human immunodeficiency virus (HIV), cannabinoid, autophagy, extracellular vesicles (EVs), transcription

## Abstract

Of the 37.9 million individuals infected with human immunodeficiency virus type 1 (HIV-1), approximately 50% exhibit HIV-associated neurocognitive disorders (HAND). We and others previously showed that HIV-1 viral RNAs, such as trans-activating response (TAR) RNA, are incorporated into extracellular vesicles (EVs) and elicit an inflammatory response in recipient naïve cells. Cannabidiol (CBD) and Δ9-tetrahydrocannabinol (THC), the primary cannabinoids present in cannabis, are effective in reducing inflammation. Studies show that cannabis use in people living with HIV-1 is associated with lower viral load, lower circulating CD16^+^ monocytes and high CD4^+^ T-cell counts, suggesting a potentially therapeutic application. Here, HIV-1 infected U1 monocytes and primary macrophages were used to assess the effects of CBD. Post-CBD treatment, EV concentrations were analyzed using nanoparticle tracking analysis. Changes in intracellular and EV-associated viral RNA were quantified using RT-qPCR, and changes in viral proteins, EV markers, and autophagy proteins were assessed by Western blot. Our data suggest that CBD significantly reduces the number of EVs released from infected cells and that this may be mediated by reducing viral transcription and autophagy activation. Therefore, CBD may exert a protective effect by alleviating the pathogenic effects of EVs in HIV-1 and CNS-related infections.

## 1. Introduction

HIV associated neurocognitive disorder (HAND) is a spectrum of disorders associated with human immunodeficiency virus type 1 (HIV-1) infection. The disorder can be classified into three different categories: asymptomatic neurocognitive impairment (ANI), mild neurocognitive disorder (MND), and the most severe form, HIV-associated dementia (HAD, sometimes referred to as AIDS Dementia complex or ADC) [1]. Since the widespread use of combined antiretroviral therapy (cART) in 1996, there was a marked reduction in the number of people with HIV (PWH) diagnosed with HAND, although the prevalence is still currently estimated to be 20–50% of HIV-1 infected individuals [2]. However, while the relative percentage of those with severe cognitive impairment has decreased, the percentage of PWH with ANI has increased. Although this trend is promising, the propensity to develop HAD is increased in individuals with ANI compared to those with normal neurocognitive function [3]. Not surprisingly, the incidence of HAND appears to be correlated with time of diagnosis and therapeutic intervention, with those treated early having the most promising prognosis and longest lifespan [4]. The inability of cART to completely suppress neurocognitive dysfunction illustrates a need for adjunct therapies that target the underlying neuropathology. 

Extracellular vesicles (EVs), nano-sized vesicles that are released from numerous cell types into different biofluids, are implicated as a potential mediator of central nervous system (CNS) dysfunction in HIV-1 patients [5,6,7,8]. During infection of the host cell, HIV-1 is able to exploit the host’s EVs and utilize them to promote viral infection, particularly through the incorporation of viral products such as RNA and proteins [5,6,9,10,11]. The packaging of viral cargo into EVs has a profound impact on recipient cells. Specifically, studies showed that in spite of antiretroviral therapy, HIV-1 TAR RNA is incorporated into EVs derived from HIV-1 infected cells in vitro and in vivo, including within EVs isolated from HIV-1 infected cerebrospinal fluid (CSF) [5,9,10,12]. These TAR-containing EVs were found to elicit increased susceptibility to infection and initiation of the NF-κB pathway through TLR3 activation leading to increased release of proinflammatory cytokines in recipient primary macrophages, potentially contributing to CNS inflammation [10]. Furthermore, several studies showed that even with suppressive antiretroviral therapy, EVs containing HIV-1 Nef, a small viral protein, were implicated in the pathogenesis of HAND [5,7,8]. EVs containing Nef induce oxidative stress in recipient neurons and promote increased expression and subsequent secretion of amyloid-beta (Aβ), suggesting that EVs can contribute to HIV-1 associated neurotoxicity [7,8].

The United Nations Office on Drugs and Crime’s 2016 World Drug Report suggests that cannabis (i.e., marijuana) is the most commonly used recreational drug worldwide, with approximately 182.5 million users across the globe. This high prevalence and recent changes in legislature for its use have led researchers to explore the potential impacts of cannabis on various disease states. Studies have indicated cannabis may indeed have therapeutic applications, particularly within the CNS [13,14,15,16,17]. Of particular interest, it is estimated that 23–56% of PWH using cannabis report therapeutic use to manage symptoms and side effects of other medications [18,19]. A study by Thames et al., which examined 55 HIV-positive individuals, demonstrated that cannabis use was associated with lower viral loads and higher CD4^+^ counts [18]. Furthermore, recent studies showed that cannabis exposure is associated with decreased odds of neurocognitive impairment in PWH and lower blood–brain barrier (BBB) permeability [20,21]. The medicinal and recreational effects of cannabis are obtained from numerous biologically active cannabinoids. Δ9-tetrahydrocannabinol (THC), the predominant psychoactive compound of cannabis, and cannabidiol (CBD), the principle non-psychoactive cannabinoid found in relatively high concentrations in Cannabis indica, are the two most commonly studied cannabinoids. Despite extensive research, relatively few studies have explored the effects of these isolated cannabinoids in the context of HIV-1 infection. Studies have suggested potential therapeutic benefits such as the inhibition of HIV-1 Tat-mediated adhesion of infected myeloid cells to extracellular matrix proteins, thereby limiting transmigration into the CNS [22]. However, additional studies are needed to further describe the therapeutic applications of specific cannabinoids during CNS HIV-1 infection and the associated neurocognitive disorders.

The current study aims to determine the mechanism by which cannabinoids such as CBD can confer neuroprotective effects during HIV-1 infection. We demonstrate that cannabinoids independently reduce the number of EVs released from HIV-1 infected myeloid cells. In addition, we observed a decrease in the copy number of HIV-1 RNAs, TAR, and genomic env packaged into EVs, suggesting that cannabinoids may have therapeutic potential in mitigating inflammation during infection.

## 2. Materials and Methods

### 2.1. Cell Culture and Reagents

U1 (HIV-1-infected promonocytic) and U937 (promonocytic) cells were cultured in complete RPMI 1640 media with 10% fetal bovine serum (FBS), 1% l-glutamine, and 1% penicillin/streptomycin (Quality Biological, Gaithersburg, MD, USA) and incubated in 5% CO_2_ at 37 °C. U1 cells were treated with varying concentrations of CBD (2-[1R-3-methyl-6R-(1-methylethenyl)-2-cyclohexen-1-yl]-5-pentyl-1,3-benzenediol; Cayman chemicals, Ann Arbor, MI, USA; Cat. #90080) and THC (Δ9-tetrahydrocannabinol; NIDA, MD, USA, Cat: 7370-023), as well as other drugs such as flavopiridol hydrochloride hydrate (Cat: F3055; Sigma Aldrich, St. Louis, MO, USA), rapamycin (Cat: Y-10219; MedChem Express, Monmouth Junction, NJ, USA), INK128 (Cat: A8551; APExBIO, Houston, TX, USA), bafilomycin A1 (Cat: 54645; Cell Signaling Technology, Danvers, MA, USA), SB203580 (Cat: HY-10256; MedChem Express), and wortmannin (Cat: W-2990; LC Laboratories, Woburn, MA, USA). U1 and U937 cells were provided by the National Institutes of Health’s (NIH) AIDS Reagent Program, Manassas, VA, USA. 

A set of primary PBMCs was purchased (Precision For Medicine, Frederick, MD, USA) and cultured in vitro first with PMA/GM-CSF for 3 days to obtain macrophages. The suspension cells were then treated with PHA/IL-2 for 3 days to obtain activated T-cells. Both macrophages and T-cells were then infected with HIV-1 89.6 strain (MOI: 10) for 6 or 12 days, respectively. The 6th-day cultures (T-cells) were then treated with cART for an additional 3 d to stop the spreading of the virus. On day 9, media was removed, and CBD was added for another 3 days. The macrophages were also treated with PMA and GM-CSF during the infection period (added every 3 days into the fresh media). On day 12 post-infection, all cells were treated with a 10 µM cocktail (Lamivudine, Tenofovir, Emtricitabine, Indinavir) and kept in culture for another 3 days. On day 15, media was removed, and CBD was added for 5 days (1, 5, 10 µM; at 0 and 48 h). All EV samples were collected using Nanotrap particles and processed for RNA analysis. 

### 2.2. Patient Samples

Plasma specimens were obtained from 15 individuals, including 10 PWH with a suppressed viral load on cART (*n* = 5 cannabis users and *n* = 5 non-cannabis users) and HIV-uninfected individuals (*n* = 5 non-cannabis users) at the Chronic Viral Illness Service/Centre for Innovative Medicine of the McGill University Health Centre (Montreal, QC, Canada). This study was ethically approved by the Research Institute of the McGill University Health Centre Research Ethics Board (MUHC 15-031/18-3835). Written informed consent was obtained before enrolment. The research was conducted in accordance with the Helsinki Declaration. 

### 2.3. ZetaView Nanoparticle Tracking Analysis (NTA)

NTA was performed with the ZetaView Z-NTA (Particle Metrix) and its software (ZetaView 8.04.02; Particle Metrix GmbH, Inning am Ammersee, Germany). The machine was calibrated according to the manufacturer’s protocol, using settings as previously described [5]. Following the measurement and removal of any outliers from the measured 11 positions, the mean size (diameter; nm) and the concentration of the sample (EV number) were analyzed by the associated ZetaView software. Measurement data from the ZetaView were used to calculate averages and standard deviations from technical triplicates using Microsoft Excel 2016.

### 2.4. EV Enrichment Using Nanotrap Particles

EVs from small volume samples were enriched using Nanotrap particles (Ceres Nanosciences, Inc; Manassas, VA, USA) as previously described [5,10,23,24,25]. Briefly, equal parts NT82 (Ceres #CN2010), NT80 (Ceres #CN1030), and 1× PBS without calcium or magnesium were combined and resuspended to create a 30% slurry. Twenty-five microliters of the NT80/82 slurry were added to 1 mL of culture supernatant and rotated overnight at 4 °C to enrich for EVs. The next day, the Nanotrap particles were pelleted, washed one time with 1× PBS without calcium or magnesium and used for further analyses.

### 2.5. RNA Isolation, Creation of cDNA, and Quantitative Real-Time PCR (RT-qPCR)

For the isolation of total RNA, cells were harvested, washed once in 1× PBS without calcium or magnesium and resuspended in 50 µL of 1× PBS. For the isolation of total RNA from EVs, Nanotrap particles were incubated and harvested as described above, then resuspended in 50 µL of 1× PBS without calcium or magnesium. Total RNA was isolated from cell pellets and NT80/82 pellets bound with EVs using Trizol Reagent (Invitrogen) as described by the manufacturer’s protocol. cDNA was generated using GoScript Reverse Transcription Systems (Promega) using Envelope Reverse: (5′-TGG GAT AAG GGT CTG AAA CG-3′; Tm = 58 °C), and TAR Reverse: (5′-CAA CAG ACG GGC ACA CAC TAC-3′, Tm = 58 °C) primers. Serial dilutions of DNA from a CEM T-cell line containing a single copy of HIV-1 LAV provirus per cell (8E5 cells) were used as the quantitative standards. cDNA samples (2 µL per well) were plated into a Master Mix (18 µL per well) containing IQ Supermix (Bio-Rad), TAR Forward Primer (5′-GGT CTC TCT GGT TAG ACC AGA TCT G-3′), TAR Reverse Primer (5′-CAA CAG ACG GGC ACA CAC TAC-3′), and TAR Probe (5′56-FAM-AG CCT CAA TAA AGC TTG CCT TGA GTG CTT C-36-TAMSp-3′). RT-qPCR conditions were as follows: one cycle for 2 min at 95 °C followed by 41 cycles of 95 °C for 15 s and 58 °C for 40 s. Reactions were performed in triplicate using the BioRad CFX96 Real-Time System. Quantitation was determined using cycle threshold (Ct) values relative to the 8E5 standard curve using the BioRad CFX Manager Software. Analysis of generated raw data was analyzed using Microsoft Excel 2016.

### 2.6. Preparation of Whole-Cell Extracts

Infected U1 cell pellets were collected, washed with 1× PBS, and resuspended in 50 µL of lysis buffer (50 mM Tris-HCl (pH 7.5), 120 mM NaCl, 5 mM EDTA, 0.5% Nonidet P-40, 50 mM NaF, 0.2 mM Na_3_VO_4_, 1 mM DTT, and 1 complete protease inhibitor cocktail tablet per 50 mL (Roche Applied Science; Philadelphia, PA, USA). The mixture was incubated on ice for 20 min with vortexing every 5 min. Following incubation, the cell debris was separated by centrifugation for 10 min at 10,000× *g*. Protein concentrations of each cell lysate were determined using the Bradford protein assay performed according to the manufacturer’s protocol (Bio-Rad).

### 2.7. Western Blot

Laemmli buffer was added to sample cell lysates (10–20 µg) or NT80/82 pellets. Lysates were heated at 95 °C for 3 min, and 15 µL of the sample was loaded onto a 4–20% Tris/glycine gel (Invitrogen; Waltham, MA, USA). When analyzing NT80/82 pellets, samples were heated at 95 °C for 3 min, vortexed, and then heated two additional times to release captured material from NT80/82 beads. Following the release of captured cargo, the total sample was then loaded onto a 4–20% Tris-Glycine gel (Invitrogen). Gels were run at 100 V and transferred onto Immobilon PVDF membranes (Millipore; Burlington, MA, USA) at 50 mA overnight. Membranes were blocked in 5% milk in PBS with 0.1% Tween-20 (PBS-T) for 2 h at 4 °C, then incubated overnight at 4 °C in PBS-T with the appropriate primary antibody: α-p24 (Cat: 4121; NIH AIDS Reagent Program), α-Nef (Cat: 3689; NIH AIDS Reagent Program), α-gp120 (Cat: 522; NIH AIDS Reagent Program), α-CD81 (Cat: EXOAB-CD81A-1; SBI, Palo Alto, CA, USA), α-VPS4 (Cat: sc-32922; Santa Cruz Biotechnology Dallas, Texas, USA), α-CHMP6 (Cat: sc-67231; Santa Cruz Biotechnology), α-Actin (ab-49900; Abcam, Waltham, MA, USA), α-CD63 (Cat: EXOAB-CD63A-1; Systems Biosciences, Palo Alto, CA, USA), α-SQSTM1/p62 (Cat: 5114; Cell Signaling Technology), α-MAP LC3 α/β (Cat: sc-398822; Santa Crux Biotechnology), α-Alix (Cat: sc-49268; Santa Cruz Biotechnology), α-ATG12 (Cat: sc-271688; Santa Cruz Biotechnology), and α-Beclin 1 (Cat: sc-48341; Santa Cruz Biotechnology). Membranes were washed with PBS-T and incubated for 2 h with the indicated HRP-conjugated secondary antibody at 4 °C. HRP luminescence was activated with Clarity or Clarity Max Western ECL Substrate (Bio-Rad) and visualized by the Molecular Imager ChemiDoc Touch system (Bio-Rad).

### 2.8. Cell Viability Assay

Cells were cultured in RPMI with 10% exosome-free FBS at 10^6^ cells per well in 6 well plates. Cells were treated with 0.005% ethanol (control), CBD (1, 5, 10, 50 µm), and THC (1, 5, 10, 50 µg per mL) once per day for 5 days. The MTT assay was used to measure cell death according to the manufacturer’s protocol (Sigma). After incubating with MTT solution for 2 h, the culture medium was aspirated, and 0.1 mL of MTT solvent was added to each well. Cell viability was determined by the formation of formazan crystal and measured by absorbance at 590 nm and 620 nm, as previously described [26]. 

### 2.9. Chromatin Immunoprecipitation (ChIP)

Cells were harvested and washed with 1x PBS at 2000× *g* for 10 min. The cells were resuspended in 1% formaldehyde, made from 37% formaldehyde solution (Cat: F1635; Sigma-Aldrich), and exosome free RPMI media supplemented with l-glutamine, penicillin/streptomycin and FBS. Samples were rocked for 30 min at room temperature and further processed using the Imprint Chromatin Immunoprecipitation Kit (Sigma). Next, the samples were sonicated, followed by an overnight 4 °C incubation while rotating with IgG or Pol II (serine 2/5) antibodies (10 µg). A 50% (*v*/*v*) protein A-Sepharose/protein G-Sepharose beads (A/G beads) (Calbiochem) was used to bind the complexes for a 2 h 4°C incubation, rotating. Samples were washed three times with 1× PBS, and proteinase K (800 units per mL) and crosslinking reversing solution (Sigma) was added for 90 min at 65 °C. DNA samples were washed and assayed for TAR DNA using qPCR analysis.

### 2.10. Kinase Assay

U1 cells (5 × 10^6^) were treated with CBD (1 µM) and THC (3.18 µM) every day for 5 days. Cells were pelleted by centrifugation at 15,000× *g* for 5 min, washed with 1× PBS, and lysed in 250 µL lysis buffer. Lysates were immunoprecipitated with 10 µg of cdk9 or IgG antibodies overnight at 4 °C, followed by a 2 h incubation with A/G beads at 4 °C. IP complexes were washed with TNE50 + 0.1% NP40 buffer and kinase buffer as described in [27], followed by 1 h incubation period at 37 °C with γ-^32^P ATP and purified histone H1. The samples (50%) were loaded and run through SDS-PAGE on a 4–20% Tris-Glycine gel, followed by staining with Coomassie blue overnight and then destained with destaining buffer (50% methanol and 10% acetic acid), and dried for 2 h. The dried gels were exposed to a PhosphorImager Cassette, followed by analysis using Molecular Dynamic’s ImageQuant Software; Los Altos, CA, USA.

### 2.11. Neurospheres 

Neural progenitor cells (NPCs; Cat: ACS-5003; ATCC, Manassas, VA, USA) were grown using STEMdiffTM Neural Progenitor Medium (STEMCELL Technologies^TM^; Vancouver, BC, Canada) and differentiated into 3D neurospheres (1 × 10^5^ cells per well) using DMEM:F12 (Cat: 30-2006; ATCC) + Neural Progenitor Cell Dopaminergic Differentiation Kit (Cat: ACS-3004; ATCC) for an incubation period of 14 d. Inverted microscopy using Zen imaging software (Zeiss; Jena, Germany) was used to develop phase-contrast images. Neurospheres were infected with HIV-1 89.6 dual-tropic strain (MOI: 10) and 10 µL of Infectin^TM^ (Virongy, LLC; Manassas, VA, USA) with a total volume of 200 µL, followed by a 48 h incubation period. Neurospheres were then gently washed with 1× PBS ± treated twice with 10 µM cocktail (Lamivudine, Tenofovir, Emtricitabine, Indinavir) ± treatment (every other day) with a titration of CBD (5 and 10 µM) for a seven-day incubation period. Neurospheres were lysed in 100 µL lysis buffer, and total lysates were run on a 4–20% Tris-Glycine SDS-PAGE, followed by Western blot assessment. For RT-qPCR analysis, RNA was isolated from neurospheres, followed by RT-qPCR analysis. 

### 2.12. Mass Spectrometry Analysis

HIV-1 infected U1 monocytes and U1 monocyte-derived macrophages (MDMs) were cultured, followed by lysis of cell pellets. Each lysate sample (250 µg) was incubated with 100 µg of D-Biotin (ThermoFisher Scientific; Cat: B1595) or biotinylated cannabidiol (KareBay Biochem, Inc., Monmouth Junction, NJ, USA), with a total volume of 800 µL, at 4 °C overnight. Streptavidin-Sepharose Beads (BioVision, Inc; Milpitas, CA, USA. Cat: 6565-2) (30 µL) were then added to each sample and incubated for 2 h at 4 °C. The beads were pelleted through centrifugation at 15,000× *g* for 5 min. The supernatants were removed, and the pellets were washed twice with TNE300 + 0.1% NP40 buffer at 15,000× *g* for 5 min. The pellets were washed with 1× PBS at 15,000× *g* for 5 min, and the supernatant was discarded. The pellets were treated with urea (8 M) and DTT (10 mM) to reduce samples, which were then alkylated with iodoacetamide (50 mM). The samples were then diluted by a solution of equal parts water and NH_4_HCO_3_ (500 mM). Samples were digested with trypsin (Promega) for 4 h at 37 °C. The sample was desalted by ZipTip, dried in SpeedVac, and then reconstituted with 10 µL of 0.1% formic acid for mass spectrometry (MS) analysis. Liquid chromatography coupled tandem mass spectrometry (LC-MS/MS) experiments were performed on an Orbitrap Fusion (ThermoFisher Scientific, Waltham, MA, USA) equipped with a nanospray EASY-nLC 1200 HPLC system. Peptides were separated using a reversed-phase PepMap RSLC 75 μm i.d. × 15 cm long with 2 μm particle size C18 LC column from ThermoFisher Scientific. The mobile phase consisted of 0.1% aqueous formic acid (mobile phase A) and 0.1% formic acid in 80% acetonitrile (mobile phase B). After sample injection, the peptides were eluted using a linear gradient from 5% to 40% B over 60 min and ramping to 100% B for an additional 2 min. The flow rate was set at 300 nL per min. The Orbitrap Fusion was operated in a data-dependent mode in which one full MS scan (60,000 resolving power) from 300 *m*/*z* to 1500 *m*/*z* was followed by MS/MS scans in which the most abundant molecular ions were dynamically selected by Top Speed and fragmented by collision-induced dissociation (CID) using a normalized collision energy of 35%. “EASY-Internal Calibration”, “Peptide Monoisotopic Precursor Selection”, and “Dynamic Exclusion” (10 s duration) were enabled, as was the charge state dependency so that only peptide precursors with charge states from +2 to +4 were selected and fragmented by CID. 

For data processing, Tandem mass spectra were searched against the NCBI human database using Proteome Discover v 2.3 from ThermoFisher Scientific. The SEQUEST node parameters were set to use full tryptic cleavage constraints with dynamic methionine oxidation. Mass tolerance for precursor ions was 2 ppm, and mass tolerance for fragment ions was 0.5 Da. A 1% false discovery rate (FDR) was used as a cut-off value for reporting peptide spectrum matches (PSM) from the database search. The mass spectrometry proteomics data were deposited to the ProteomeXchange Consortium via the PRIDE [28] partner repository with the dataset identifier PXD027263.

### 2.13. Pathway Enrichment Analysis

Proteins with ≥2 peptide hit were included for enrichment analysis. Peptide hit filtered lists were used for the removal of non-specific interactions by comparing D-Biotin to CBD-Biotin protein hits. Reactome pathway enrichment analysis was performed using an online tool named “g:Profiler” to determine pathways associated with proteins spectra retrieved from mass spectrometry [29]. For enrichment analysis, protein accession numbers were fed as an input with a cut off threshold of 0.05 and run-on g:SCS algorithm. g:SCS method is the default method for computing multiple testing correction for *p*-values gained from GO and pathway enrichment analysis, corresponds to an experiment-wide threshold of a = 0.05, i.e., at least 95% of matches above the threshold are statistically significant. a is the already set probabilty cut off value of 0.05. As a result, enriched pathways are shown as a bar graph between the Reactome pathways identification number/name and negative *p*-value.

### 2.14. Densitometry Analysis

ImageJ software was used to conduct densitometry analysis on the kinase and Western blot images. Background measurements were deducted from each lane to account for exposure, followed by normalization of each lane to each respective actin measurement. The control lane (lane 1) was set to 100% to demonstrate a concise increase or decrease for the subsequent lanes. Percentage changes were calculated by subtracting normalized lane measurements from the normalized control lane.

### 2.15. Statistical Analysis

Standard deviations were analyzed using Microsoft Excel software for quantitative experiments. Two-tailed Student’s *t*-test was used to calculate *p*-values, where analysis was determined statistically significant when * = *p* < 0.05, ** = *p* < 0.01, and *** = *p* < 0.001.

## 3. Results

### 3.1. Cannabidiol Inhibits the Release of EVs from HIV-1 Infected Monocytes

**Figure 1:**
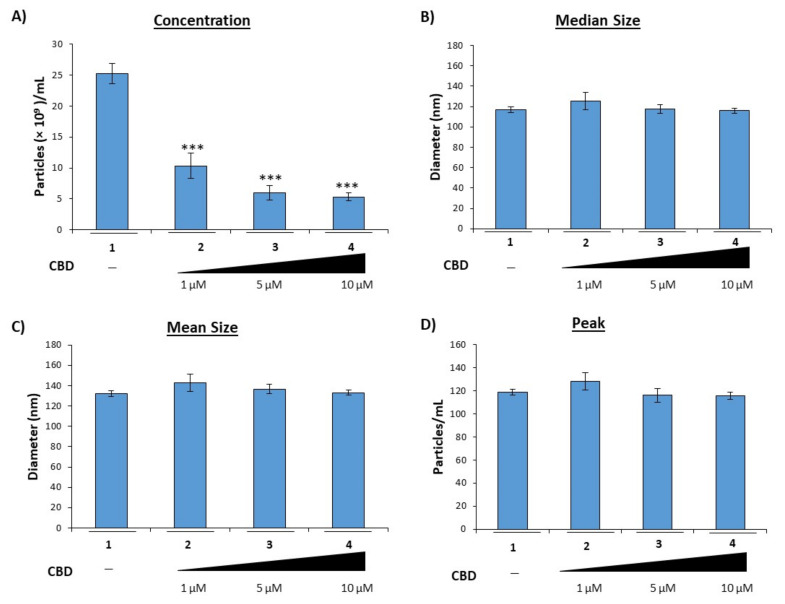
CBD lowers EVs released from HIV-1 infected monocytes. HIV-1 infected U1 monocytic cells (1 × 10^6^) were treated with a titration of CBD (1, 5, and 10 µM) every day for 5 days. Cells were pelleted, and supernatant was used for Zetaview NTA analysis to determine (**A**) EV concentration, (**B**) median size, (**C**) mean size, and (**D**) peak size. Each bar represents an average of three independent replicates. Student’s *t*-test was used for statistical analysis, where *** = *p* < 0.001.

We and others previously showed that EVs released from virally infected cells contain viral products that contribute to viral pathogenesis, specifically chronic inflammation in those with long-term infections such as HIV-1 [5,10,30]. Furthermore, we showed that EVs released from infected cells contain harmful viral proteins and RNAs despite treatment with HIV-1 specific antiretrovirals as well as general antivirals such as interferon-α [5], suggesting a need for improved therapies which target EV-mediated mechanisms in HIV-1 infection. Therefore, we reasoned that CBD, which was previously found to have significant anti-inflammatory properties [31], could mitigate the enhanced inflammation observed in PWH through modulation of EV release from infected cells. To this end, HIV-1 infected monocytes (U1) were treated twice with a titration of CBD over 5 days with no effects on cell viability (Supplementary Figure S1A). Titration of CBD on U1 infected cells showed similar results compared to control U937 cells (Supplementary Figure S1B). Next, supernatants from infected cells treated with CBD were harvested and analyzed using ZetaView NTA. The data in Figure 1A suggest that treatment with CBD results in a significant reduction in the number of EVs released from virally infected cells; specifically, treatment with CBD (lanes 2–4) resulted in a 44%, 76%, and 79% decrease in EVs, respectively, as compared to an untreated control. 

To verify that the observed decrease in EVs was not the result of modifications in packaging of various cargos into larger EVs, the mean diameter size (Figure 1B), median diameter size (Figure 1C), and peak diameter size (Figure 1D) were also analyzed. Treatment with any of the concentrations of CBD resulted in little to no change in diameter. Collectively, these results suggest that CBD can potentially reduce the overall release of EVs.

### 3.2. Cannabidiol Decreases EV-Associated Viral Proteins and Viral RNAs

**Figure 2:**
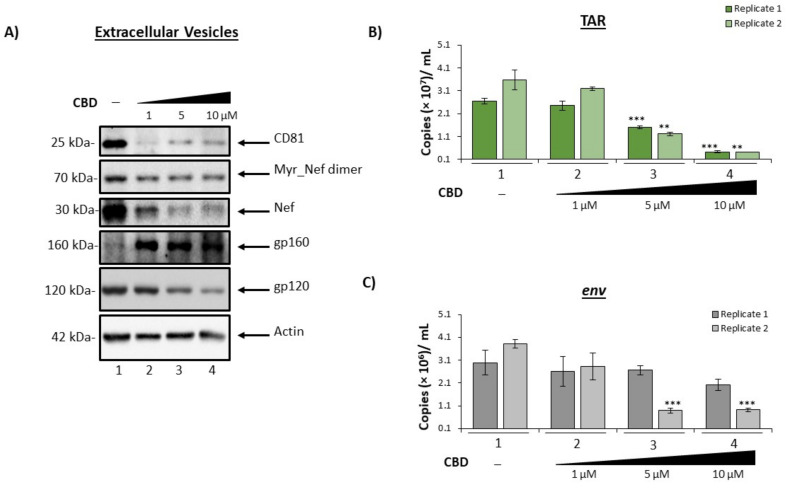
CBD decrease the amount of viral cargo carried inside EVs released from infected monocytes. U1 cells (1 × 10^6^) were treated with a titration of CBD (1, 5, 10 µM) every day for 5 days. Cellular supernatant was collected, exosomes were enriched with nanoparticles (NT80/82) beads overnight at 4°C, (**A**) pelleted, run through SDS-PAGE, and Western blotted for exosomal (CD81) and HIV viral markers (myr-Nef, Nef, gp120), as well as actin. (**B**) Following the same experimental design as (**A**), cellular supernatant was nanotrapped and pelleted. RNA was isolated from the samples, followed by RT-qPCR analysis for HIV-1 viral transcripts, TAR and (**C**) env. Student’s *t*-test was used for statistical analysis, where ** = *p* < 0.01, and *** = *p* < 0.001.

We have previously shown that despite cART, EVs released from HIV-1 infected cells contain viral proteins and RNA that can elicit detrimental effects in uninfected recipient cells [5,6,9,10]. To verify that cannabinoid treatment not only results in a reduction in EV number, but also a reduction in the presence of viral products, cells were treated twice with a titration of CBD and incubated for 5 days. Following incubation, supernatants were collected, and EVs were enriched using NT80/82 particles, which was previously shown to be effective in enriching EVs from low volume culture supernatant [5,10,23,24,25,32]. Enriched samples were then analyzed by Western blot for the presence of EV marker and HIV-1 viral proteins. The data in Figure 2A show a reduced level of CD81, a tetraspanin protein marker associated with exosomes when treated with CBD (lanes 2–4). The most drastic decrease in CD81 expression was achieved by low titer CBD which resulted in an 80% reduction as indicated by densitometry analysis (lane 2; Supplementary Figure S2A).

The viral protein Nef is reported to be incorporated into EVs from numerous cell types, including astrocytes and microglia. Further evidence identified Nef as a potential contributor to HIV-1 pathogenesis through various mechanisms, including the promotion of secreted proinflammatory cytokines, the suppression of neuronal action potentials, and disruption of the BBB [5,7,33,34,35]. Data in Figure 2A illustrate a dose-dependent decrease in EV-associated Nef (30 kDa) and its membrane-associated myristoylated Nef dimer (70 kDa) when treated with CBD. Densitometry analysis shown in Supplemental Figure 2B,C suggests there is a more drastic decrease in the lower molecular weight form of Nef (up to 79%, lane 3; Supplementary Figure S2C) as compared to the myristoylated dimer (up to 31%, lane 3; Supplementary Figure S2B). 

Gp120 can contribute to enhanced HIV-1 infection of the CNS and the corresponding neuroinflammation through disruption of the BBB, increased cytokine secretion, specifically TNFα via activation of microglia and astrocytes, and increased neuronal sensitivity to calcium fluctuation [36,37,38,39,40,41,42,43]. Numerous studies have shown the potential of endogenous cannabinoids, or endocannabinoids, to alleviate gp120-induced neuroinflammation in HIV-1 infection [44]. Therefore, we reasoned that CBD, which targets the same receptors as endocannabinoids, could also result in a reduction in EV-associated gp120. Results in Figure 2A show a reduction in EV-associated gp120 ranging from 39–58% (Supplementary Figure S2D; lanes 3–4) with CBD treatment, suggesting a potential to mitigate the gp120-induced neuropathogenesis. 

HIV-1 infection also leads to the incorporation of viral RNAs into EVs released from infected cells. HIV-1 TAR RNA is a short, non-coding RNA produced in infected cells as a result of non-processive transcription. We and others have found TAR RNA incorporated into EVs released from infected cells and can be detected in several biofluids at relatively high copy numbers [5,9,10,12]. TAR within EVs was shown to increase viral pathogenesis through the down-regulation of apoptosis, increased susceptibly to HIV-1 infection, and activation of TLR3 to subsequently stimulate the production of pro-inflammatory cytokines within uninfected recipient cells [9,10]. To determine if CBD possessed the potential to limit the incorporation of such RNAs into EVs, the same supernatants were enriched for EVs using NT80/82 particles, total RNA was isolated and subjected to RT-qPCR for the presence of a short non-coding HIV-1 RNA (TAR), and viral full-length genomic RNA (env). The results in Figure 2B show a dose-dependent decrease in the presence of EV-associated TAR RNA following the addition of CBD (lanes 2–4). All doses of CBD elicited a reduction in TAR RNA as compared to the control (lane 1). A statistically significant decrease in TAR RNA within EVs was observed in the two highest concentrations, which produced an average of 55%, and 86% reduction, respectively, from two replicates. Similarly, treatment with CBD resulted in the reduction of env RNA within EVs (Figure 2C). Taken together, these results indicate that CBD can decrease the number of EVs released from HIV-1 infected cells, which leads to a decrease in the amounts of viral products (protein and RNA) available to contribute to chronic inflammation.

**Figure 3:**
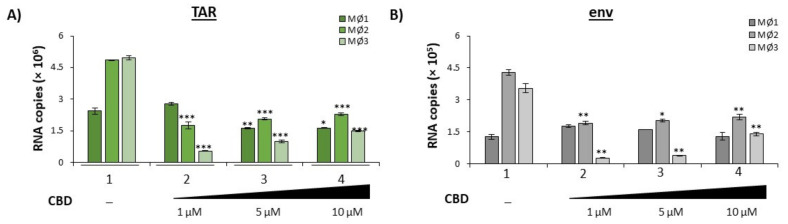
CBD lowers viral RNAs in EVs released from HIV-1 infected primary macrophages. Primary PBMCs were purchased (Precision For Medicine, Frederick, MD, USA) and cultured in vitro first with PMA/GM-CSF for 3 days to obtain macrophages, and were then infected with HIV-1 89.6 strain (MOI: 10) for 12 days. The macrophages were treated with PMA and GM-CSF during the infection period (added every 3 days into the fresh media). On day 12, all cells were treated with cART (4 antivirals) and kept in culture for another 3 days. On day 15, media was removed, and CBD was added for 5 days (1, 5, 10 µM; at 0 and 48 h). Supernatants were then isolated, and exosomes were enriched using NT80/82 beads overnight at 4 °C. The NT80/82 beads were then pelleted, RNA was isolated, followed by RT-qPCR analysis for (**A**) TAR and (**B**) env viral RNA transcripts. Data shown are an average of 3 replicates. Student’s *t*-test was used for statistical analysis, where * = *p* < 0.05, ** = *p* < 0.01, and *** = *p* < 0.001.

To examine whether the same trend could be observed in a primary cell model, three independent HIV-1 infected primary macrophage cell cultures were treated with a titration of CBD. EVs were enriched from the extracellular supernatant using NT80/82 beads and analyzed for HIV-1 TAR and env (Figure 3). Data in Figure 3A demonstrate a relationship between increasing doses of CBD treatment and a decrease in TAR RNA within EVs from all three infected primary macrophages. Additionally, a similar trend is observed with the amount of env RNA in secreted EVs released from two out of three infected primary macrophages (Figure 3B). A similar trend was observed in EVs from HIV-1 infected primary macrophages treated with cART and CBD (Supplementary Figure S3A,B), and EVs from HIV-1 infected primary T-cells treated with CBD alone (Supplementary Figure S4A,B).

We also focused on using THC as a control and asked whether similar effects of CBD could also be observed when applied to infected cells. We titrated THC and performed zeta view analysis, Western blot and RT/qPCR analysis for EVs concentration, tetraspanin/viral protein markers, and viral RNA, respectively. Data indicated that there was a drop in EVs concentrations (Supplementary Figure S5A–D), CD81, and Nef protein levels (Supplementary Figure S6A) and viral RNAs (Supplementary Figure S6B,C). Interestingly, the greatest reduction of Nef (30 kDa) was achieved by administration of CBD, whereas the greatest reduction of myristoylated Nef dimer (70 kDa) was elicited by THC, potentially suggesting two different mechanisms of EV cargo modification. 

Additionally, both cannabinoids elicited a decrease in the presence of the processed envelope glycoprotein, gp120, suggesting that both cannabinoids could potentially interfere with the proteolytic cleavage of HIV-1 precursor polyproteins, potentially through the modulation of intracellular Ca^2+^ levels as the primary endoprotease responsible for cleavage is a calcium-dependent enzyme [45]. Therefore, CBD and/or THC may also contribute to the levels of EVs containing unprocessed glycoprotein and/or defective viral particles. 

To apply these findings to in vivo HIV-1 infection, total EVs were enriched from plasma samples from 15 individuals; 5 healthy donors, 5 HIV-1 positive donors, and 5 HIV-1 positive donors who used cannabis were trapped using NT80/82 particles and analyzed via Western blot for the presence of HIV-1 Nef protein. The results in Supplementary Figure S7 show a reduction in the presence of Nef protein in EVs (possibly from CBD component of cannabis) isolated from HIV-1 infected individuals who used cannabis. These results confirm that the cannabinoid CBD has the potential to decrease the release of EVs containing viral products from infected primary cells. 

### 3.3. Cannabidiol Inhibit HIV-1 Transcription in Monocytes

**Figure 4:**
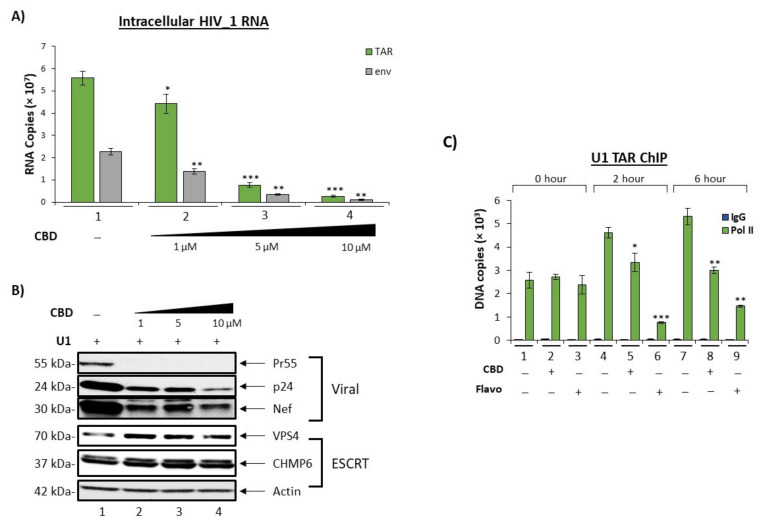
CBD lowers intracellular viral transcription and downstream viral products. U1 cells (1 × 10^6^) were treated with a titration of CBD (1, 5, 10 µM) every day for 5 days. (**A**) Intracellular RNA was isolated and analyzed through RT-qPCR for HIV-1 viral transcripts TAR and env. Each bar represents an average of three independent replicates. (**B**) U1 cells (2 × 10^6^) were treated with a titration of CBD (1, 5, 10 µM) every day for 5 days. Cells pellets were lysed, run through SDS-PAGE, and Western blotted for EV biogenesis marker VPS4, CHMP6, viral markers (Pr55, Nef, p24), and actin. (**C**) U1 cells (5 × 10^6^) were treated ± CBD (10 µM) and Flavopiridol hydrochloride (50 nM; indicated as Flavo) and incubated for the following time points: 0 min, 2 h, and 6 h. Cells were cross-linked with 1% formaldehyde solution and ChIP-ed for IgG and Pol II. ChIP DNA samples were analyzed via qPCR for TAR and env. Student’s *t*-test was used for statistical analysis, where * = *p* < 0.05, ** = *p* < 0.01, and *** = *p* < 0.001.

Given that our results indicate a reduction in EV release and EV-associated HIV-1 viral RNAs and proteins, we hypothesized that the observed reduction could be the result of changes in intracellular viral transcription resulting in a decreased production of viral products observed in initially released EVs. Therefore, U1 monocytes were treated with a titration of CBD every day for 5 days. Total RNA was isolated from the harvested cell pellets followed by RT-qPCR analysis of three independent replicates for TAR and env RNAs. Both TAR and env viral RNAs exhibited a dose-dependent decrease in response to CBD treatment (Figure 4A; lanes 2–4 vs. lane 1), with the highest dose resulting in a 95% and 96% decrease as compared to the untreated control. This may indicate that the reduction in secreted EV-associated viral TAR and env RNAs may be the result of CBD-mediated transcription inhibition. Furthermore, the reduction in both TAR and full length genomic (env) RNA suggest that CBD has the potential to inhibit viral transcription at the level of transcription initiation.

To verify that the CBD-mediated transcription inhibition resulted in a decrease in the production of viral proteins within HIV-1 infected cells, U1 cells were treated with a titration of CBD every day for 5 days and intracellular lysate was used for Western blot analysis of specific viral proteins, Nef, Pr55, and p24. As expected, intracellular levels of Nef, Pr55, and p24 proteins were downregulated post-CBD treatment in a dose-dependent manner (Figure 4B). The most significant reduction in all three viral proteins, Pr55 (99%), p24 (76%), and Nef (62%), was observed with the highest concentration of CBD (Supplementary Figure S8A–C). This is potentially due to innate differences in the abundance of these mRNAs. Overall, there were typically fewer gag (Pr55/p24) mRNA transcripts within HIV-1 infected cells as compared to nef mRNAs, which account for nearly 50% of all HIV-1 doubly spliced RNA in infected cells [46].

EVs consist of different subpopulations of vesicles that vary in size, biomarker characterization, and functionality [47,48,49]. Previously, we showed that one of the mechanisms by which exosomes (an EV subpopulation) are released, the endosomal sorting complexes required for transport (ESCRT) pathway, is altered when HIV-1 infected cells are treated with combination antiretroviral therapy (cART) drugs [5]. Briefly, the ESCRT pathway involves a series of interactions between four different ESCRT complexes (-0, -I, -II, -III), which contain a specific set of proteins to recruit exosomal cargo and promote intraluminal vesicular (ILV) budding inside multivesicular bodies (MVBs), as well as a VPS4 complex which disassociates ESCRT complexes post exosomal formation [50]. The observed decrease in CD81 levels, an exosomal biomarker, as a result of CBD treatment and the decrease in EV concentration (Figure 1 and Figure 2), suggest that the ESCRT pathway may be involved in the CBD-mediated changes in EV release and cargo. Western blot analysis of ESCRT proteins showed a slight increase in CHMP6, a protein involved in the ESCRT-III complex, and VPS4, a protein involved in exosomal membrane scission, indicating that the later stages of EV maturation may not be the target of CBD (Figure 4B).

CBD-mediated transcription inhibition was further explored by investigating RNA polymerase II (Pol II) loading activity in comparison to a known transcription inhibitor, Flavopiridol (Flavo), which was shown to inhibit cyclin-dependent kinase 9 (cdk9)/Cyclin T1 complex activity [51,52,53,54]. U1 monocytes were treated with CBD or Flavo for short periods (0, 2 and 6 h) due to the fact that HIV-1 transcription activity is a rapid event that can be scored using chromatin immunoprecipitation (ChIP) assay for transcription factor occupancy. Treated samples were lysed and incubated with IgG control and Pol II (serine 2/5) antibodies for ChIP, followed by qPCR analysis for TAR DNA. Data in Figure 4C show minimal TAR DNA for all of the IgG ChIPs, which served as a negative control. As expected, there was no change in the amount of TAR DNA pulled down with Pol II with any treatment compared to the untreated control at 0 h samples (lanes 1–3). However, both the 2 h and 6 h Pol II pulldown samples showed statistically significant decreases of Pol II-associated TAR DNA when treated with CBD or Flavo in comparison to the untreated samples (lanes 4–6 and lanes 7–9), with Flavo exhibiting more effective inhibition in comparison to CBD. It is also important to note that CBD did not inhibit cdk9/Cyclin T1 activity in vitro, indicating that lower Pol II loading onto DNA may not be related to cdk9/Cyclin T1 kinase activity (data not shown). Collectively, these data indicate that CBD has a partial effect on HIV-1 transcription and Pol II loading. This data emphasizes the potential inhibitory nature of CBD (either direct or indirect) on serine 2/5 phosphorylated Pol II activity of HIV-1 viral transcription in monocytes.

### 3.4. Cannabidiol Alters Intracellular and Secretory Autophagy Pathways

**Figure 5:**
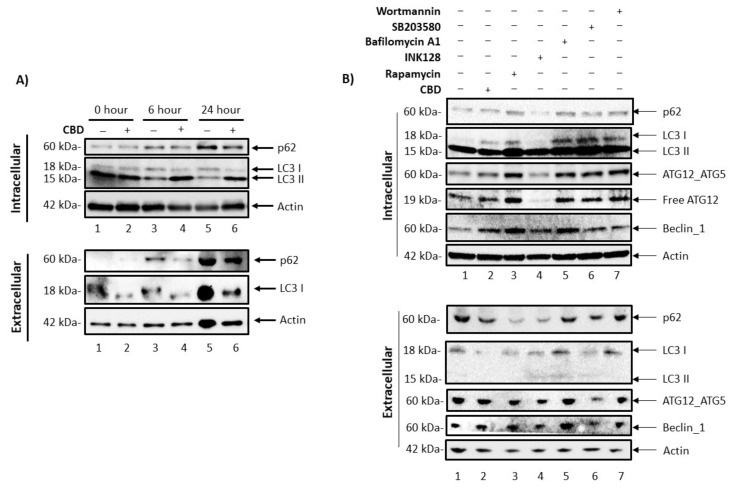
CBD promotes autophagy turnover, resulting in a decrease in extracellular autophagosomal secretion. (**A**) U1 cells (1 × 10^6^) were treated ± CBD (10 µM) for the following incubation periods: 0, 6, 24, and 48 h. Cells were pelleted and lysed, exosomes from supernatants were enriched with NT80/82 beads overnight at 4 °C. Samples were run through SDS-PAGE, and Western blotted for autophagy proteins (p62 and LC3) and Actin. (**B**) U1 cells (1 × 10^6^) were treated ± 10 µM CBD (every day), rapamycin (50 nM), INK128 (50 nM), bafilomycin A1 (50 nM), SB203580 (20 µM), wortmannin (2 nM) for a 5-day incubation period. Cells were pelleted and lysed, exosomes from supernatants were enriched using NT80/82 beads overnight at 4 °C. Samples were run through SDS-PAGE, and Western blotted for autophagy proteins (p62, LC3, ATG12-ATG5, ATG12, Beclin-1), and actin.

We previously described a complex relationship between the intracellular autophagy pathway, secretory autophagy pathway, and EVs, where regulation of the autophagy pathway in infected cells can result in the blocking of viral protein degradation and, in turn, the export of viral products through EVs [55]. Based on this, we next asked whether CBD had any effect on EV release by examining the secretory autophagy pathway. In order to evaluate the levels of intracellular autophagy proteins in HIV-1 infected cells, U1 cells were treated with CBD for 0, 6, and 24 h, followed by Western blot analysis for autophagosomal markers, p62 and LC3 I/II (Figure 5A). The data indicates that, over time, there was an increase in autophagosomal p62 production in the untreated samples (lanes 1, 3, and 5). Conversely, a 50% decrease of intracellular p62 with CBD treatment was observed at the 24 h (lane 5 vs. lane 6) timepoint. The normalized densitometry results of intracellular p62 are shown in Supplementary Figure S9. This decrease in SQSTM1/p62 protein, an autophagosome cargo adaptor protein that is degraded upon fusion of the autophagosome with the lysosome, suggests an increased autophagic flux in response to CBD treatment. A decrease in LC3 I levels were also observed in CBD treated samples up to 24 h as compared to the untreated control, suggesting an increased conversion of LC3 I to LC3 II, which is indicative of elongation of the pre-autophagosomal membrane and is critical for the initiation of autophagy. This is further supported by the higher levels of LC3 II in CBD treated samples that are sustained at 6- and 24 h as compared to their respective controls, further indicating active autophagosome formation in CBD treated samples. In order to examine any alterations that CBD might have on secretory autophagy in HIV-1 infected monocytes, EVs were enriched from the supernatants using NT80/82 and analyzed by Western blot for changes in secreted autophagosome proteins. As expected, there was an increase in the levels of extracellular p62 in untreated controls (Supplementary Figure S9D, lanes 1, 3, 5), suggesting an increase in the number of secreted autophagosomes over time as a result of virally mediated autophagy inhibition observed in the intracellular protein levels. This is in line with numerous studies which showed that the HIV-1 viral proteins Tat and Nef are involved in autophagy deregulation in infected cells [56,57]. Furthermore, lower p62 levels were observed with CBD treatment from the 6 h time point onwards compared to the untreated controls (lanes 3, 5 vs. 4, 6, respectively), suggesting an overall decreased secretion of autophagosomes from HIV-1 infected cells. Additionally, there was an overall lower level of LC3 I in EVs secreted from CBD treated cells (lanes 3, 5 vs. 4, 6, respectively). The actin normalized densitometry analysis is shown in Supplementary Figure S9. Taken together, decreased p62 and LC3 levels in both intracellular and in secreted autophagosome vesicles suggests that CBD may activate the autophagy pathway, thereby lowering overall secretory autophagy. 

To determine the specific points at which CBD inhibits intracellular and extracellular autophagosome formation and secretion, we compared CBD treatment with known inhibitors of various steps in the autophagy pathway. Cell viability was assessed among a panel of titrated autophagy drugs, and the least toxic concentration per drug was utilized for further analysis (Supplementary Figure S10). Post-titrations, we then treated U1 cells with CBD (10 µM) every day, rapamycin (50 nM), INK128 (50 nM), bafilomycin A1 (50 nM), SB203580 (20 µM), or wortmannin (2 nM) for 5 days. Our rationale for using these drugs was that they regulate different steps of autophagy, including nucleation inhibition by inhibiting MTOR activity (Rapamycin, INK128) or by inhibiting pre-autophagosomal VPS34 complex activity (Wortmannin, SB203580) and autophagosome-lysosome fusion inhibition (Bafilomycin A1) [58,59,60,61]. Therefore, a side-by-side comparison to CBD would potentially allow for a better definition of which step of autophagosome formation is primarily regulated by CBD. U1 cells were treated with the autophagy regulators and harvested along with their corresponding supernatants for further analysis using Western blot against p62, LC3, ATG12-ATG5 complex, free ATG12, Beclin-1, and actin. Results in Figure 5B (intracellular proteins, top panel) show autophagy protein profiles associated with the various autophagy regulators. Interestingly, the autophagy protein profile resulting from treatment with CBD most closely reflected the profile exhibited by cells treated with the autophagy inducers, rapamycin and INK128 (lanes 2–4), as indicated by the low levels of LC3 I and ATG12-ATG5 complex, and by high levels of Beclin-1. The corresponding supernatants were enriched for EVs and also analyzed for the presence of autophagy proteins. Treatment with CBD, rapamycin, and INK128 resulted in a reduction of EV-associated p62 and LC3 I levels (Figure 5B, lower panel, lanes 2–4). Conversely, treatment with autophagy inhibitors, bafilomycin A1, SB203580, and wortmannin (Figure 5B, lower panel, lanes 5–7) resulted in sustained levels of EV-associated p62, elevated levels of LC3 I and -II as compared to the untreated, HIV-1 infected control (lane 1). Little to no change in EV-associated levels of ATG12-ATG5 complex and Beclin-1 was observed with any treatment. The Actin normalized densitometry analysis is shown in Supplementary Figure S11. In order to summarize, a quantitative summary of the change in EVs, viral Proteins and RNA is given in Table 1. Furthermore, we investigated the relationship between CBD and autophagy using a biotinylated-CBD pull-down from U1 monocytes and monocyte-derived macrophages for Mass spectrometry analysis. Tandem mass spectra were searched against the NCBI human database using Proteome Discover, and proteins with ≥2 peptide hit were included for analysis. The background non-specific interactions were removed by comparing D-Biotin to CBD-Biotin protein hits. The protein accession number from the filtered list were fed into “g:Profiler” with a cut off threshold of 0.05 for Reactome pathway enrichment analysis. The results from enrichment analysis showed significant enrichment of proteins in Reactome autophagy pathways (Macrophage: R-HSA-9663891, R-HSA-1236974, R-HSA-1632852, R-HSA-9612973; Monocyte: R-HSA-9646399, R-HSA-9663891, R-HSA-9612973, R-HSA-1632852, R-HSA-9613829, Supplementary Figure S14). Collectively, these data indicate that CBD may promote activation of the autophagy pathway, potentially at the level of upstream mTOR signaling and/or pre-autophagosome formation. This is also supported by studies in several tissues, including mesenchymal stem cells, endothelial cells, neurons, as well as in vitro and mouse models of Parkinson’s and Alzheimer’s Disease [62,63,64,65,66]. 

### 3.5. Cannabidiol Reduces Production of Viral Products in a 3D Neurosphere Model

In recent years, three-dimensional (3D) culture systems have emerged as reliable in vitro tools for studying not only normal developmental processes but also disease modeling and drug discovery. Generally speaking, the 3D architecture provides a more tissue-like environment in which cell-to-cell and cell-to-matrix interactions are expected to more closely mimic the in vivo phenotype [67]. Additionally, these cultures may express more biologically relevant responses to diffusible factors, such as drugs of abuse.

With respect to the CNS, induced pluripotent stem cells (iPSCs) are widely used to study several different neuropathologies ranging from neurodegenerative disorders to neurotrophic viruses [68,69,70,71,72,73,74,75]. More recently, others have utilized iPSC-derived neural progenitor cells (NPCs) to generate 3D neurospheres that are susceptible to viral infection, including Zika virus and Herpes Simplex Virus 1 [76,77,78]. For these reasons, we chose to utilize a similar platform to potentially model HIV-1 infection as well as the effects of treatment with both antiretroviral compounds and CBD.

**Figure 6:**
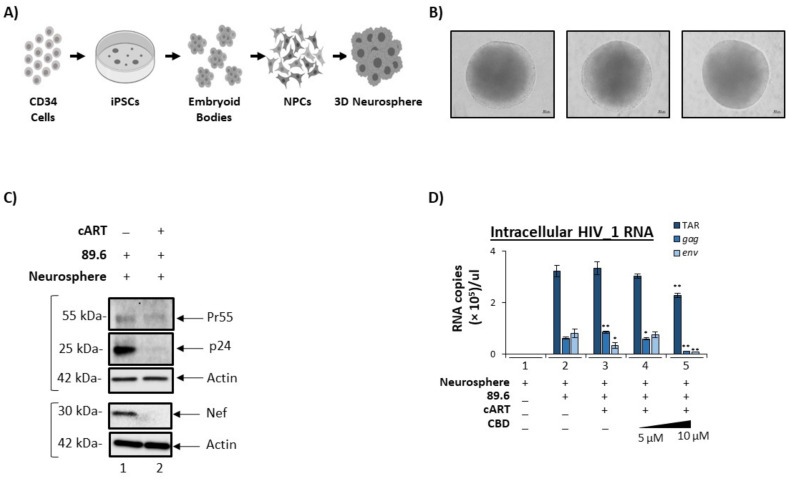
CBD lowers viral transcripts in 3D neurospheres. (**A**) Differential workflow of 3D neurospheres. (**B**) Phase-contrast microscopic images of three 3D neurospheres taken where scale bar = 100 µm. (**C**) 3D neurospheres were treated with HIV-1 89.6 (MOI: 10) viral strain ± cART (10 µM; Tenofovir, Emtricitabine, Lamivudine, and Indinavir), incubated for 7–14 days, harvested and lysed. Samples were run through SDS-PAGE and Western blotted for viral proteins (Pr55, p24, Nef and Actin). For Nef Western blot, samples were run on a different gel and also probed for actin. (**D**) 3D neurospheres were treated ± 89.6 viral strain ± cART (10 µM; Tenofovir, Emtricitabine, Lamivudine, and Indinavir Lamivudine,) ± CBD titration (5 and 10 µM) and incubated for 7 days. RNA was isolated from the 3D neurospheres and supernatants, followed by RT-qPCR analysis for viral RNA transcripts TAR, TAR-gag, env. Student’s *t*-test was used for statistical analysis, where * = *p* < 0.05, ** = *p* < 0.01.

Here, commercially available NPCs were used to establish neurospheres for infection. The diagram in Figure 6A provide a high-level summary of the workflow involved in this process, which utilized CD34^+^ cells as the original starting material to generate iPSCs, followed by embryoid body formation, and then differentiation into NPCs. To induce neurosphere formation, approximately 1 × 10^5^ NPCs were individually seeded in U-shaped wells. After approximately 48 to 72 h of incubation, NPCs had self-aggregated into well-defined neurospheres and were subsequently cultured in the presence of a differentiation medium for an additional two weeks. Representative images in Figure 6B show the appearance of three individual neurospheres, which clearly demonstrate their uniform shape and structure. Importantly, our previous research has suggested that differentiated neurospheres are composed of a mixture of neurons (dopaminergic, gabaergic, glutamatergic), glial cells (astrocytes and microglia-like), and NPCs. We recently published a more extensive description of this 3D system followed by infections [79].

To determine if neurospheres were susceptible to HIV-1 infection, differentiated neurospheres were cultured with HIV-1 dual tropic 89.6 (MOI: 10) with or without the addition of cART cocktail (10 µM; Tenofovir, Emtricitabine, Lamivudine, and Indinavir) for a period of seven days. Neurospheres were then harvested for downstream experiments. To evaluate the extent of viral replication, Western blot was performed to assess the relative expression levels of HIV-1 viral proteins. As shown in Figure 6C, expression of Pr55, p24, and Nef proteins was detected in neurospheres cultured with HIV-1 89.6 (lane 1). In contrast, the presence of cART resulted in a reduction in all three viral proteins, with the most drastic decrease being observed in p24 (92%) and Nef (98%). These data were validated via densitometry analysis by normalizing expression levels to actin (Supplementary Figure S12). Overall, this data suggests that NPC-derived neurospheres can harbor replicating HIV-1 and, furthermore, highlights the efficacy of cART in a 3D model.

Next, the levels of intracellular viral RNA transcripts were assessed to determine the effects of CBD treatment on HIV-1 infected neurospheres. Prior to these experiments, the expression of relevant receptors in neurospheres was confirmed, including cannabinoid receptors CB1 and CB2, as well as the serotonin receptor 5-HT1A and the vanilloid receptor, which were previously found to bind cannabinoids [80] (Supplementary Figure S13). We then performed RT-qPCR analysis to quantify the expression of TAR, gag, and env. Data in Figure 6D show that upon cART treatment, there was a significant decrease in gag and env RNA relative to HIV-1 89.6 alone (lanes 2 vs. 3), potentially suggesting that cART treatment of neurospheres lowers only full length genomic viral RNAs while not affecting short non-coding RNAs. Interestingly, treatment of HIV-1 infected neurospheres with CBD resulted in a significant reduction of all three viral transcripts (Figure 6D, lanes 3 vs. 5).

These data suggest that CBD in combination with cART may be more effective at reducing viral transcription than cART alone. In conclusion, these data emphasize the potential of CBD for mitigating the inflammation and neuronal degradation associated with HIV-1. 

## 4. Discussion

Numerous studies implicated EVs as a mechanism of disease pathogenesis, including viral infections [10,24,81,82,83,84]. In long-term HIV-1 infections, several HIV-1 proteins and RNAs were found in the CSF of HIV-1 infected individuals, potentially owing to increased protein half-life through modifications inhibiting their reuptake [12,85] and encapsulation in EVs such as exosomes [5]. As a result of their increased half-life, viral proteins accumulate in the extracellular space within the CNS and as well as traverse the BBB via absorptive endocytosis to elicit neuroinflammation through interactions with microglia, astrocytes, and neurons. Although cART is effective for suppressing viremia, a shift in the neurocognitive symptoms with existing regimens indicates potential changes in neuropathogenic mechanisms in treated patients. This, coupled with the fact that these proteins and RNAs continue to be produced despite cART suggests a gap in current cART regimens which implies the need for the development of new therapeutics with high CNS penetrance designed to degrade or inhibit soluble/EV-associated viral proteins and RNAs.

In this study, we have investigated the use of cannabinoid CBD as a potential EV-modulator (Figure 1). The highly lipophilic nature of cannabinoids makes them ideal candidates for the treatment of EV-associated neurological complications of infection, particularly HIV-1 associated neurocognitive deficits. Previous work from our lab and others showned that the short non-coding viral RNA, TAR, which is the result of non-processive viral transcription, can be found within cells latently infected with HIV-1 and can be released in EVs [5,9,10,86,87,88]. Additionally, TAR RNA associated with EVs was also shown to effectively bind TLR3 and subsequently activated NF-κB to elicit and increase pro-inflammatory cytokines, thereby contributing to chronic inflammation in long-term HIV-1 patients, specifically those with HIV-1 associated neurological conditions [10]. The potential of EV-associated TAR RNA to activate TLR3 was recently confirmed by Chen et al., who found that TAR RNA within EVs could enter cells to promote the development of non-AIDS-defining cancers via activation of the ERK cascade [89]. The reduction of both viral proteins and RNA (Figure 2 and Figure 3) by low dose CBD points to the widespread potential of CBD to combat inflammation in HIV-1 patients both within the CNS and peripherally. This is in line with studies that showed an association between lower counts of CD16^+^ monocytes (which facilitate entry of the HIV-1 virus into the CNS) and the use of cannabis [90,91,92]. The inhibition of EV release from circulating monocytes and immune-competent cells of the CNS, such as microglia, perivascular macrophages, and pericytes, could potentially mitigate the levels of chemokine and cytokine released from these cells as various proinflammatory molecules were found to be associated with EVs. This diminished release of inflammatory factors would result in a decrease in the activation of astrocytes, which serve as expanders of neuroinflammation in HIV, numerous other infections, and several CNS-related diseases.

While decades of research led to great advances in the treatment of HIV-1, allowing for adequate suppression of the virus, improved quality of life, and a longer life span, there is currently no HIV-1 transcription inhibitor included in antiretroviral therapies. This gap allows for persistent HIV-1 transcription with occasional full length read through which can result in the production of viral proteins and fully infectious viruses. The presented data suggest CBD has the potential to inhibit HIV-1 transcription (Figure 3). Interestingly, a comparison study with THC as a control indicated that two cannabinoids likely have different mechanisms by which they alter viral transcription, with CBD likely acting at the level of transcription initiation and THC acting on transcription elongation (THC data not shown). These findings are supported by differences in the transcriptional regulation of cellular genes in myeloid cells by CBD and THC [93,94,95]. These studies found CBD to increase the expression of negative regulators of transcription factors NF-kB and AP-1, both of which can bind the HIV-1 promoter. Further research is needed to explore CBD-mediated transcription regulation (direct or indirect) during chronic viral infections.

Viruses have evolved mechanisms by which they can hijack numerous host cell pathways to promote pathogenesis. HIV-1 accessory proteins such as Nef, Tat, and Vif were shown to inhibit nearly all stages of autophagy, including nucleation, sequestration, elongation of the phagophore, and autophagosome-lysosomal fusion for the purpose of preventing host cell-mediated degradation of viral products [55,96]. The inhibition of the cell degradation pathway induces the accumulation of the excess host cellular products, as well as the accumulation of the viral RNAs and proteins in the cytosol. We showed that through CBD-mediated activation of the intracellular autophagy pathway (Figure 5), the host cell degradation pathway continues to degrade viral proteins and RNAs, which may contribute to the lowered incorporation of viral proteins and RNAs into EVs (seen in Figure 2 and Figure 3) thereby limiting HIV-1 pathogenesis. Along these lines, a recent publication showed that CBD induces autophagy in a concentration-dependent manner that requires crosstalk between the extracellular signal-regulated protein kinases 1 and 2 (ERK1/2) and AKT, also known as protein kinase B (PKB). These signaling pathways are essential for control proliferation, cell survival and growth [97].

When the relationship between CBD and autophagy was investigated using a biotinylated-CBD pull-down from U1 monocytes and monocyte-derived macrophages for spectrometry analysis, Reactome pathway enrichment analysis showed significant enrichment of proteins in Reactome autophagy pathways (Macrophage: R-HSA-9663891, R-HSA-1236974, R-HSA-1632852, R-HSA-9612973; Monocyte: R-HSA-9646399, R-HSA-9663891, R-HSA-9612973, R-HSA-1632852, R-HSA-9613829, Supplementary Figure S14). Our lab recently published a study demonstrating the reduction of latency-reversing agent (LRA)-mediated production of proinflammatory EVs released from HIV-1 infected cells by two autophagy inducers, rapamycin and INK128, exhibiting a correlation between inflammation and autophagy [98]. Furthermore, other studies showed that HIV-1-mediated autophagy deregulation was shown in neurons and dendritic cells, with increased secretory autophagosomes shown in HAND patients exhibiting encephalitis [99]. Overall, this demonstrates the correlation between HIV-1 mediated autophagy inhibition in the CNS and neuroinflammation, as well as propagates the potential for CBD to combat this inflammation.

Numerous receptors were found to mediate the actions of CBD and/or THC, including the cannabinoid receptors, CB1 and CB2, both of which are Class A G protein-coupled receptors that are established to bind endogenous cannabinoid ligands, most notably N-arachidonoylethanolamine (anandamide or AEA) and 2-arachidonoylglycerol (2-AG) [100]. Additional studies showned peroxisome proliferator-activated receptor-γ (PPARγ) [101,102,103,104,105], 5-HT1A receptor [106,107,108,109], adenosine A2A receptor [110,111,112], G protein-coupled receptor 55 (GPR55) [113], G protein-coupled receptor 18 (GPR18) [114], and transient receptor potential cation channel subfamily V member 1 TRPV1 receptor [115,116] to mediate some CBD actions within the CNS. Interestingly, capsaicin activation of the TRPV1 receptor elicited an increase in the production of exosomes as measured by an increase in tumor susceptibility gene 101 (TSG101) and flotillin in EV preps from dorsal root ganglia neurons [117], suggesting the inhibitory effect of CBD may be cell-type and/or ligand-specific.

Few studies utilized 3D organoids to study systematic pathogenesis progression within particular organs. More specifically, the development of CNS organoids is increasingly crucial for the examination of neurodegenerative diseases, such as HAND, amyotrophic lateral sclerosis, and multiple sclerosis, as invasive surgeries for CNS are less than optimal for living patients exhibiting neurodegeneration. Our study showed the expression of endocannabinoid receptors in the 3D neurospheres, which implies the promising potential for CBD and/or THC drug delivery (Supplementary Figure S13). Additionally, successful infection of the 3D neurosphere and lowering of its viral products by CBD indicates the potential CBD has in lowering EV-associated viral products in a systematic HIV-1 infected organoid model (Figure 6). It is important to note that the high density and tight junctions formed throughout the neurosphere may affect CBD uptake to certain regions of the neurosphere, which may potentially contribute to the low efficacy of low concentration of CBD treatment observed in altering viral transcripts. Although more research is needed to examine the characteristics, viral infection, and drug uptake on the 3D neurospheres, the impact of CBD-mediated lowered viral transcription in neurospheres (Figure 6) combined with CBD-mediated lowered incorporation of viral RNAs in EVs from HIV-1 infected patients undergoing cannabis treatment (Supplementary Figure S7) implies the potential for cannabinoid-induced lowering of neuroinflammation seen in HAND.

Although this study focuses on EVs released from HIV-1 infected cells, these findings can be applied to any EV-mediated dysfunction. Numerous studies suggested that CBD’s primary therapeutic value lies in its ability to reduce inflammation [118]. Along these lines, several groups identified the presence of cytokines on the surface of and encapsulated in EVs, suggesting that EVs play a key role in numerous diseases by mediating an inflammatory response [119,120,121]. The statistically significant reduction in EV release suggests that cannabinoids, particularly CBD, exert their anti-inflammatory effects by limiting EV release, thereby broadening the application of these findings not only to other infectious diseases but also to any diseases with an inflammatory component.

## 5. Conclusions

**Figure 7:**
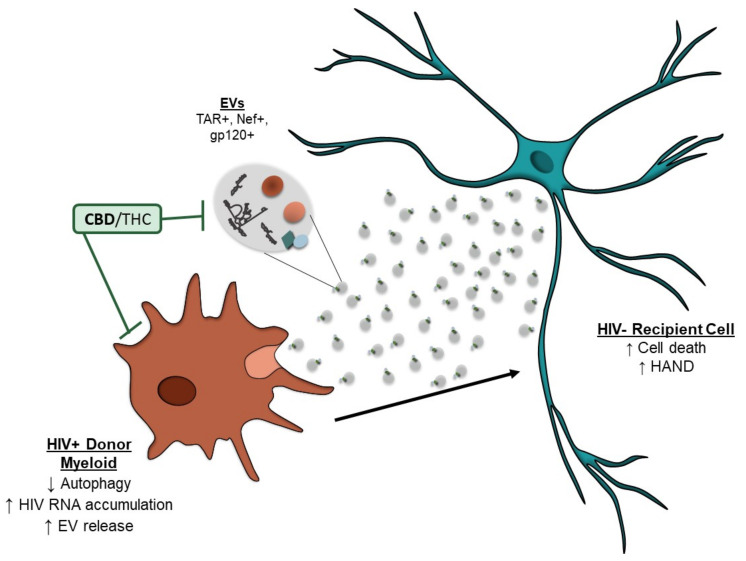
Summary of effects of cannabinoids on HIV-1 infected myeloid cells and their EVs. HIV-1 infected myeloid cells, even in the presence of cART, can transcribe viral RNAs that can be made into proteins. HIV proteins or potentially RNA can alter the autophagy pathway, resulting in the accumulation of viral products in the cytosol. This can induce increased secretion of EVs carrying viral RNAs and proteins, which can potentially contribute to the neuroinflammation seen in HAND and neuronal cell death. Cannabinoids, CBD can inhibit the abundance of EVs secreted from HIV-1 infected myeloid cells, as well as the viral RNA and protein cargo incorporated inside the EVs potentially through inhibition of intracellular transcription processes and restoration of the autophagy pathway. This can contribute to decreased EV-mediated inflammation and cellular death.

Despite cART, myeloid lineage cells have persistent viral transcription, which can lead to the production of viral proteins. HIV-1 proteins and potentially RNA can alter the autophagy pathway, which causes an accumulation of viral products in the cytoplasm, contributing to cellular stress. To alleviate the stress, viral RNA and proteins can be released in EVs, which can contribute to the inflammation associated with HAND. This research shows that cannabinoids (i.e., CBD) are effective in reducing the number of EVs released from HIV-1-infected cells. The cannabinoids also decreased the amount of EV-associated RNA and protein incorporated into EVs, potentially through the regulation of viral transcription and restoration of the autophagy pathway (Figure 7). These findings suggest cannabinoids may be beneficial in the treatment of virus-associated inflammation. 

## 6. Patents

Fatah Kashanchi; Catherine DeMarino. Intracellular Modulation of Extracellular Vesicle Release, US Patent PCT/US19/56453, 16 November 2019. Licensed to Targeted Pharmaceuticals LLC. 

## Figures and Tables

**Table 1:** (Summary) Effect of cannabidiol (CBD) on HIV-1 infected cells and released EVs.

			% Change
**Extracellular Vesicles (EVs) Release**	EVs Characteristics	Concentration Size	↓79% −
	EV-Associated Viral Proteins	Nef (30 kDa) Myristoylated Nef dimer (70 kDa) Gp120	↓79% ↓31% ↓58%
	EV-Associated Viral RNAs	TAR env	↓55–86% ↓33–75%
	EV-Associated Autophagy Proteins	p62 LC3 I	↓50% ↓46%
**Intracellular Effects**	HIV-1 Transcription *	TAR env	↓95.4% ↓95.7%
	HIV-1 Proteins	Pr55 p24 Nef	↓99% ↓76% ↓62%
	ESCRT (Endosomal Sorting Complexes Required for Transport Pathway) Proteins	VPS4 CHMP6	− −
	Autophagy Proteins	p62 LC3 I LC3 II	↓53% ↓72% ↑53%

The percentage (%) change in protein and RNA amount was estimated using Densitometry analysis and RNA copies from RT/qPCR, respectively. The % changes are shown as a number with symbols as no change “−“, decrease “↓”, and increase “↑”. * Similar effects were seen in 3D Neurospheres.

## Data Availability

The mass spectrometry proteomics data is available via ProteomeXchange with identifier PXD027263.

## Supplementary Materials

The following supporting information can be downloaded at: https://www.mdpi.com/article/10.3390/cells11040723/s1, Figure S1: Cell viability analysis of CBD on HIV-1 infected monocytes; Figure S2: Normalized densitometry analysis of CBD lowering of EV-associated viral cargo from HIV-1 infected monocytes; Figure S3: CBD lowers EV-associated viral RNAs from HIV-1 infected primary macrophages treated with cART; Figure S4: CBD lowers EV-associated viral RNAs from HIV-1 infected primary T-cells; Figure S5: THC lower EVs released from HIV-1 infected monocytes; Figure S6: THC decrease the amount of viral cargo carried inside EVs released from infected monocytes; Figure S7: CBD lowers EV-associated viral proteins from HIV-1 infected individuals; Figure S8: Normalized densitometry analysis of CBD lowering intracellular viral cargo in HIV-1 infected monocytes; Figure S9: Normalized densitometry analysis of CBD treatment of HIV-1 infected monocytes over time and autophagy; Figure S10: Cell viability analysis of autophagy drugs on HIV-1 infected monocytes and secreted EVs; Figure S11: Normalized densitometry analysis of CBD and autophagy drug treatment on HIV-1 infected monocytes; Figure S12: Normalized densitometry analysis of cART on HIV-1 infected 3D neurospheres; Figure S13: Expression of glial receptors in 3D neurospheres; Figure S14: Mass spectrometry analysis of biotinylated CBD pull down from HIV-1 infected lysates.

## Abbreviations

HAND	HIV-associated neurocognitive disorders
HAD	HIV-associated dementia
cART	Combined antiretroviral therapy
PWH	People with HIV
CNS	Central nervous system
CSF	Cerebrospinal fluid
BBB	Blood–brain barrier
THC	Δ9-tetrahydrocannabinol
CBD	Cannabidiol
TAR	Trans-activation response element
ESCRT	Endosomal sorting complexes required for transport
iPSCs	induced Pluripotent stem cells
pol	Polymerase
3D	Three-dimensional
