# Supplementary material for: Cannabinoids Reduce Extracellular Vesicle Release from HIV-1 Infected Myeloid Cells and Inhibit Viral Transcription
Source: Cells. 2022 Feb 18;11(4):723. doi: 10.3390/cells11040723 (PMC8869966; doi:10.3390/cells11040723)
Supplement: Supplementary file 1 [file cells-11-00723-s001.zip › Supplementary Information DeMarino et al. Proof Read.pdf]

Supplementary Information

Cannabinoids reduce extracellular vesicle release from HIV-1 infected myeloid cells and inhibit viral transcription

Catherine DeMarino[1†,] Maria Cowen[1†,] Pooja Khatkar[1†,] Bianca Cotto[2], Heather Branscome[1], Yuriy Kim[1], Sarah Al Sharif[3], Emmanuel T Agbottah[4],Weidong Zhou[5], Cecilia T Costiniuk[6,7], Mohammad-Ali Jenabian[8], Cohava Gelber[9], Lance Liotta[5], Dianne Langford[2] and Fatah Kashanchi[1,*]

[1] Laboratory of Molecular Virology, School of Systems Biology, George Mason University, Manassas, VA, USA
[2] Department of Neuroscience, Lewis Katz School of Medicine at Temple University, Philadelphia, PA, USA
[3] Department of Clinical Laboratory Sciences, College of Applied Medical Sciences, King Saud Bin Abdulaziz University for Health Sciences; King Abdullah International Medical Research Center, Jeddah, Saudi Arabia
[4] Montgomery College, Takoma Park, Maryland, USA.
[5] Center for Applied Proteomics and Molecular Medicine, George Mason University, Manassas, VA, USA
[6] Chronic Viral Illness Service, McGill University Health Centre, Montreal, QC, Canada
[7] Research Institute of McGill University Health Centre, Montreal, QC, Canada
[8] Department of Biological Sciences, University of Quebec at Montreal (UQAM), Montreal, QC, Canada
[9] Serpin Pharma, Manassas, VA, USA
* Correspondence: to whom correspondence should be addressed.
† authors contributed equally

* Correspondence: Fatah Kashanchi (fkashanc@gmu.edu)

Supplementary Information

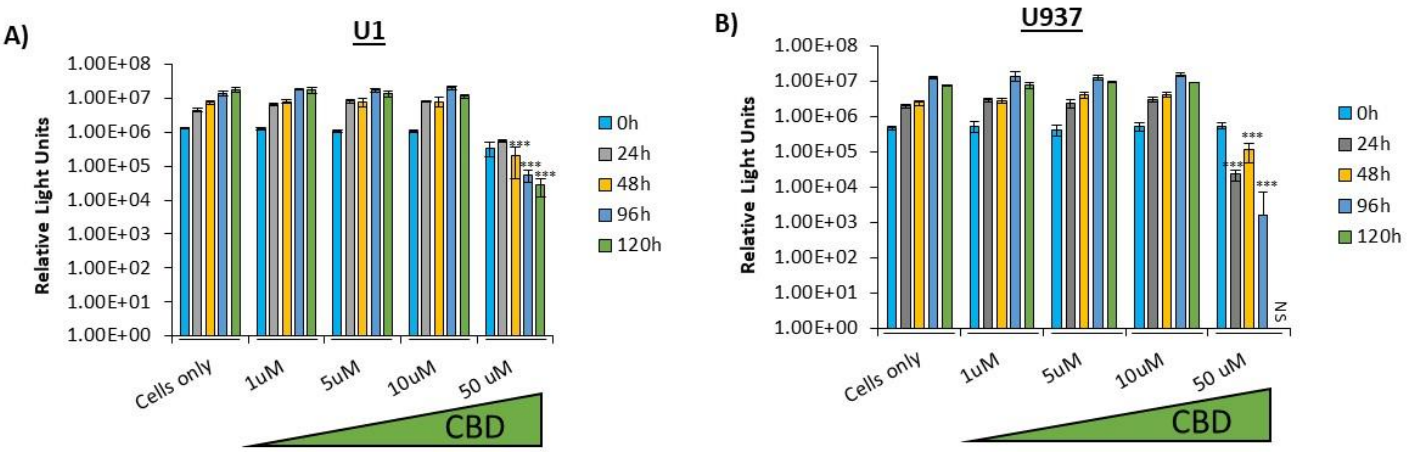

Figure S1. Cell viability analysis of CBD on HIV-1 infected monocytes. HIV-1 infected U1 monocytic cells (5 x 10^4) and uninfected U937 monocytic cells (5 x 10^4) were treated with CBD at (1, 5, 10 and 50 µM) concentration for 5 days at 0h and 48h timepoints. Cell viability was determined using the MTT assay at 0h, 24h, 48h, 96h and 120h timepoints. No light signal (NS) was detected in U937 cells with 50 µM concentration at 120h timepoint. Student's *t*-test was used for statistical analysis, * p-value ≤ 0.05; ** p-value ≤ 0.01, *** p-value ≤ 0.001.

Supplementary Information

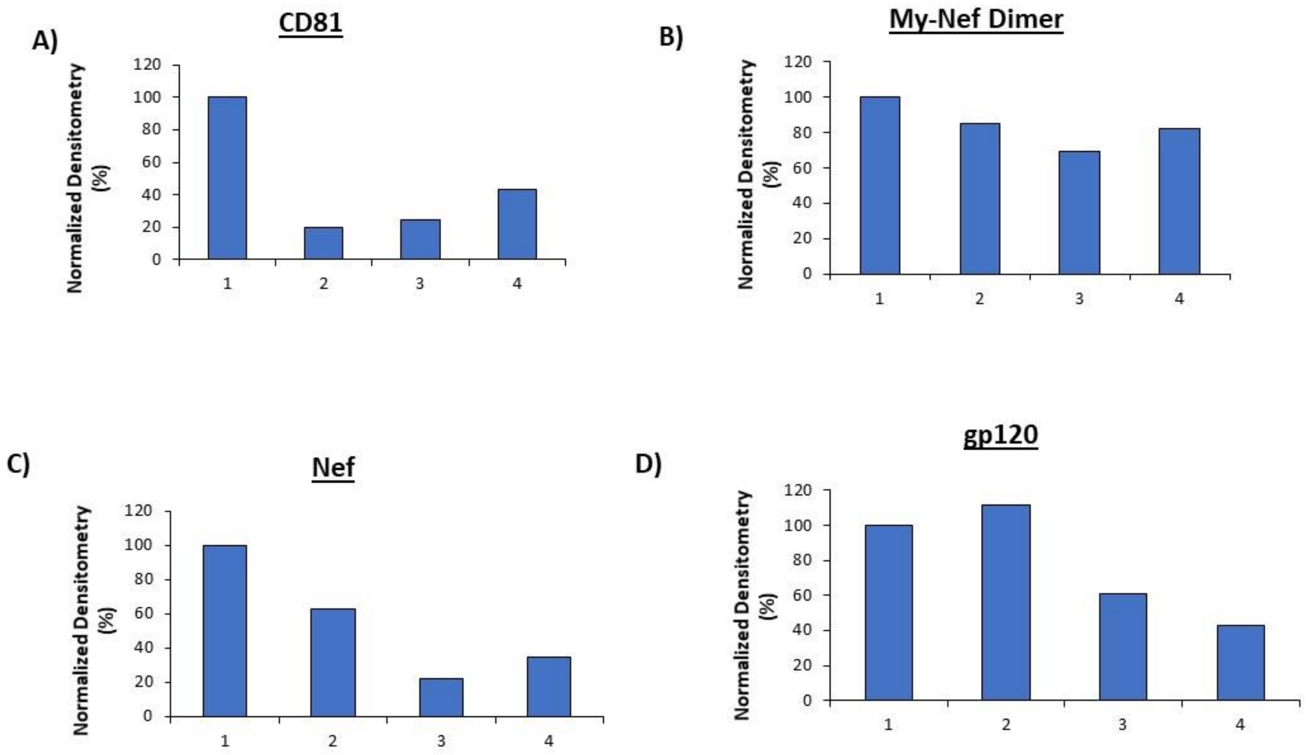

Figure S2. Normalized densitometry analysis of CBD lowering of EV-associated viral cargo from HIV-1 infected monocytes. (**A**) CD81, (**B**) Myr-Nef, (**C**) Nef, (**D**) and gp120 densitometry counts were performed on the Western blot data from Figure 2A, all of which were normalized to Actin levels.

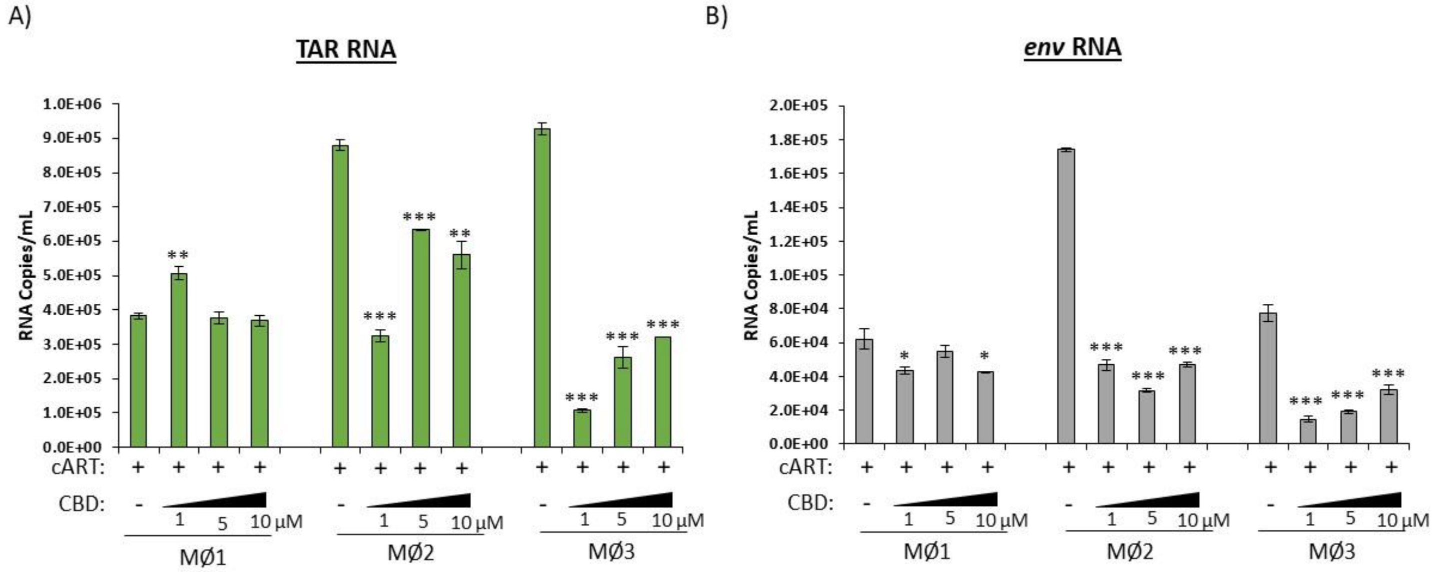

Figure S3. CBD lowers EV-associated viral RNAs from HIV-1 infected primary macrophages treated with cART. HIV-1 infected primary macrophages (1 x 10^6 cells) were treated with cART (10 µM; Emtricitabine, Tenofovir, Darunavir and 5 µM Ritonavir) twice for 5 days. EVs from the supernatant were enriched using NT80/82 beads and RNA was isolated, followed by RT-qPCR analysis for (A) TAR and (B) *env* RNA transcripts in three biological replicates. Student's *t*-test was used for statistical analysis, where * p-value ≤ 0.05; ** p-value ≤ 0.01, *** p-value ≤ 0.001.

Supplementary Information

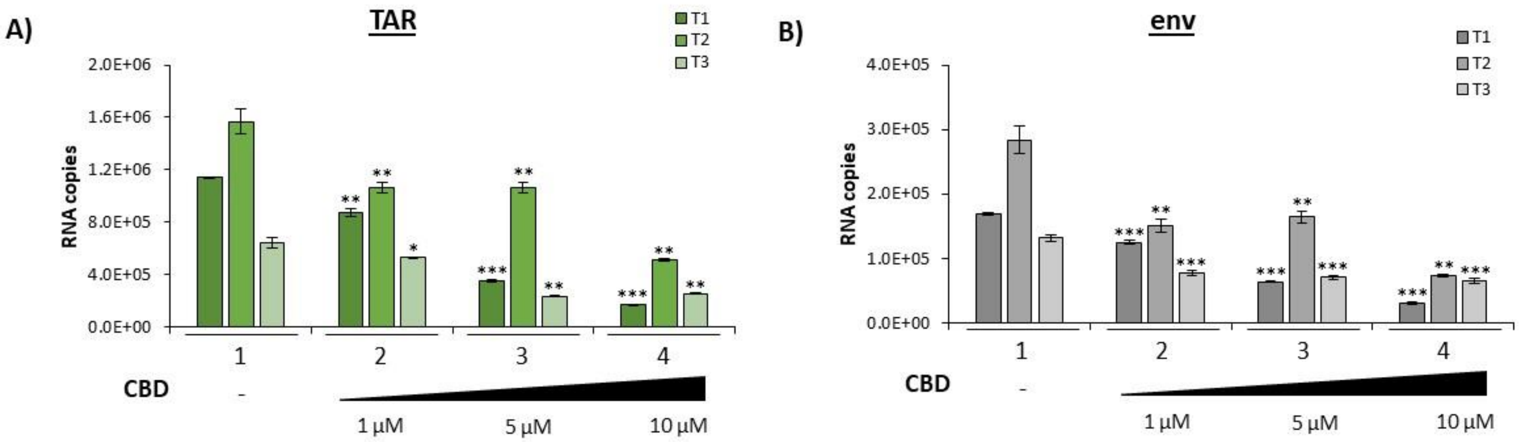

Figure S4. CBD lowers EV-associated viral RNAs from HIV-1 infected primary T-cells. PBMCs were purchased and cultured *in vitro* first to obtain macrophages. The suspension cells were then taken and treated with PHA/IL-2 for 3 days to obtain T-cells, and were then infected with HIV-1 89.6 strain (MOI:10) for 6 days. The 6th-day cultures were then treated with cART for an additional 3 days to stop the spreading of the virus. On day 9, media was removed and CBD (1, 5, 10 µM) was added for another 3 days. EVs from the supernatant were enriched using NT80/82 beads, and RNA was isolated from the EVs. RT-qPCR analysis was performed for presence of (A) TAR and (B) *env* viral RNA transcripts in three biological replicates. Student's *t*-test was used for statistical analysis, where * p-value ≤ 0.05; ** p-value ≤ 0.01, *** p-value ≤ 0.001.

Supplementary Information

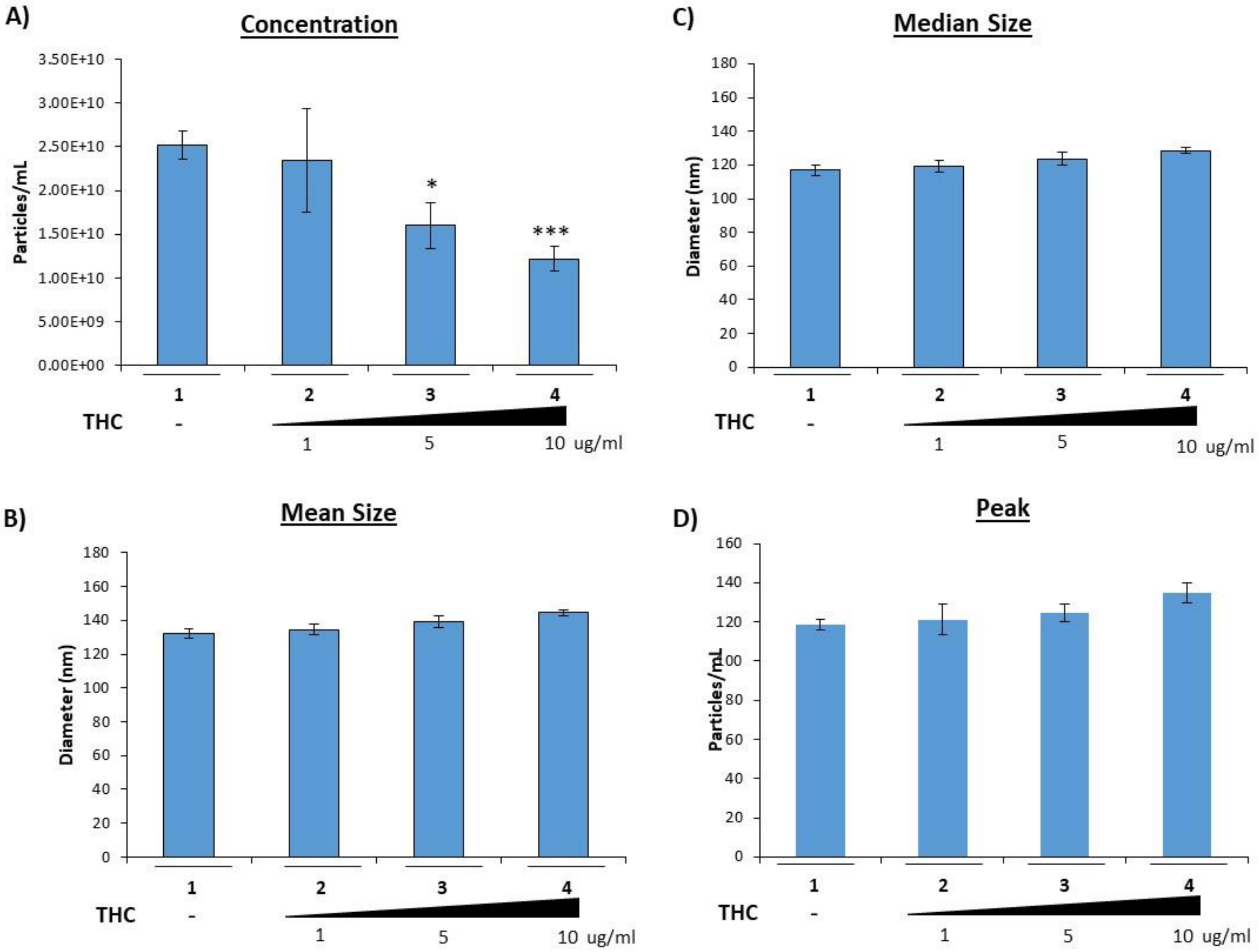

Figure S5. THC lower EVs released from HIV-1 infected monocytes. HIV-1 infected U1 monocytic cells (1 x 10^6) were treated with a titration of THC (1, 5, and 10 µg/mL) every day for 5 days. Cells were pelleted and supernatant was used for Zetaview NTA analysis to determine (**A**) EV concentration, (**B**) median size, (**C**) mean size, and (**D**) peak size. Each bar represents an average of three independent replicates. Student's *t*-test was used for statistical analysis, where * p-value ≤ 0.05; ** p-value ≤ 0.01, *** p-value ≤ 0.001.

Supplementary Information

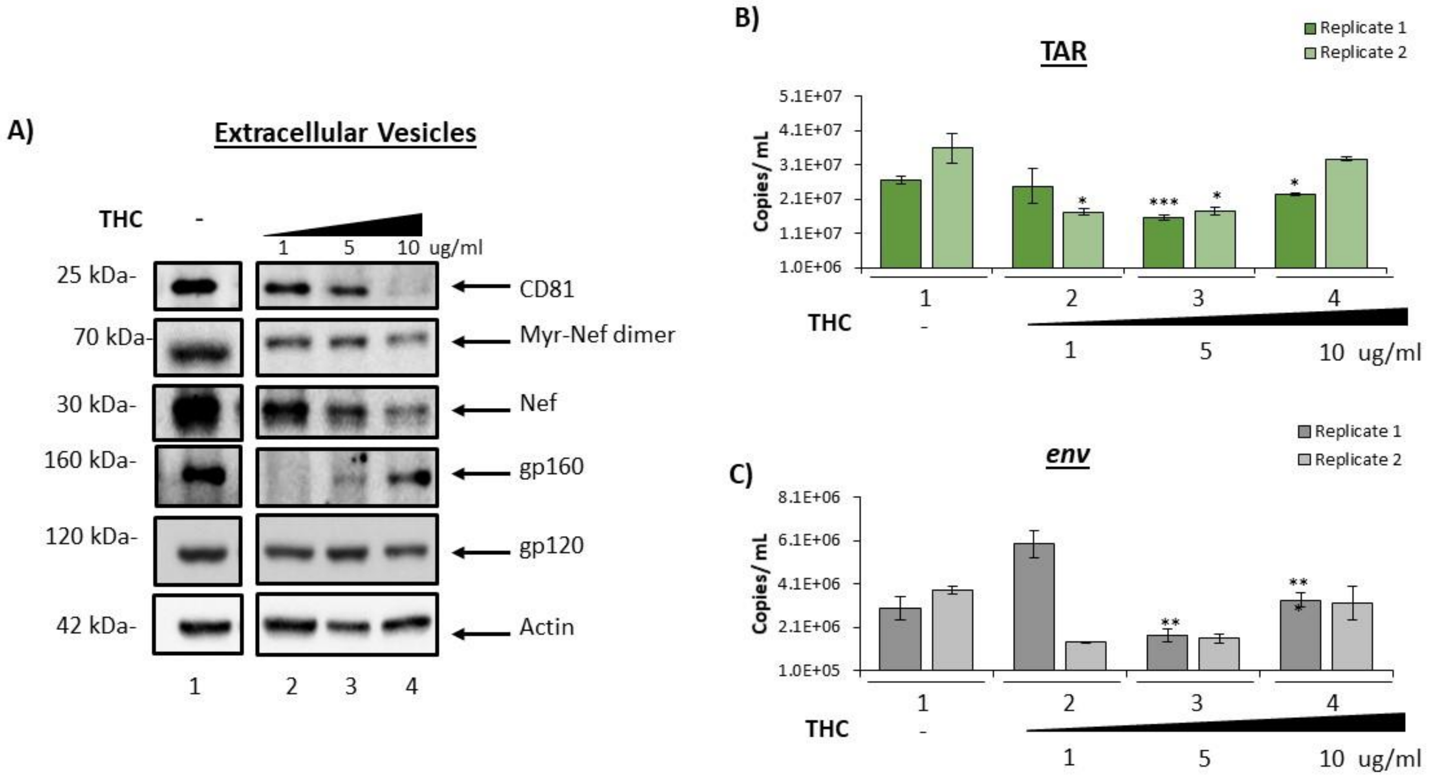

Figure S6. THC decrease the amount of viral cargo carried inside EVs released from infected monocytes. U1 cells (1 x 10^6) were treated with a titration of THC (1, 5, 10 µg/mL) every day for 5 days. Cellular supernatant was collected, exosomes were enriched with nanoparticles (NT80/82) beads overnight at 4°C, (**A**) pelleted, run through SDS-PAGE, and western blotted for exosomal (CD81) and HIV viral markers (myr-Nef, Nef, gp120), as well as actin. **B**) Following the same experimental design as (A), cellular supernatant was nanotrapped and pelleted. RNA was isolated from the samples, followed by RT-qPCR analysis for HIV-1 viral transcripts, TAR and (**C**) *env*. Student's *t*-test was used for statistical analysis, where * p-value ≤ 0.05; ** p-value ≤ 0.01, *** p-value ≤ 0.001.

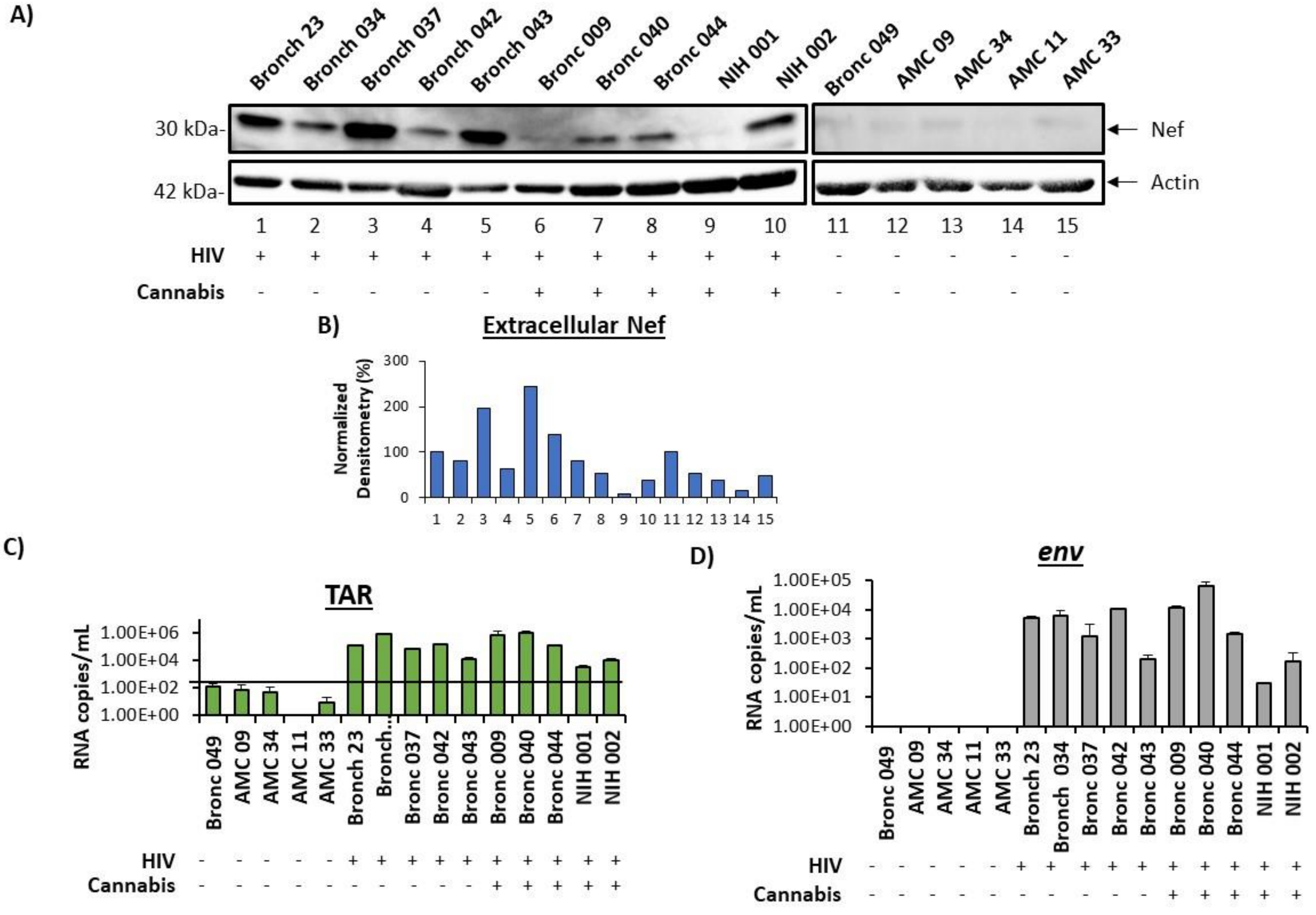

Figure S7. CBD lowers EV-associated viral proteins from HIV-1 infected individuals. **A**) Plasma from three groups of patients (HIV-1 infected, HIV-1 infected taking cannabis, uninfected) were extracted. The EVs from the plasma samples were enriched using NT80/82 particles, and were run through SDS-PAGE, followed by Western blot analysis for Nef and Actin. **B**) Densitometry counts of Nef protein levels from panel A were calculated, normalized to Actin, and plotted. **C**) Following the same experimental design as panel A, RNA was isolated from EVs enriched by NT80/82 particles of the patient biofluids, followed by RT-qPCR analysis for levels of TAR and (**D**) *env* RNA transcripts. Student's *t*-test was used for statistical analysis, where * p-value ≤ 0.05; ** p-value ≤ 0.01, *** p-value ≤ 0.001.

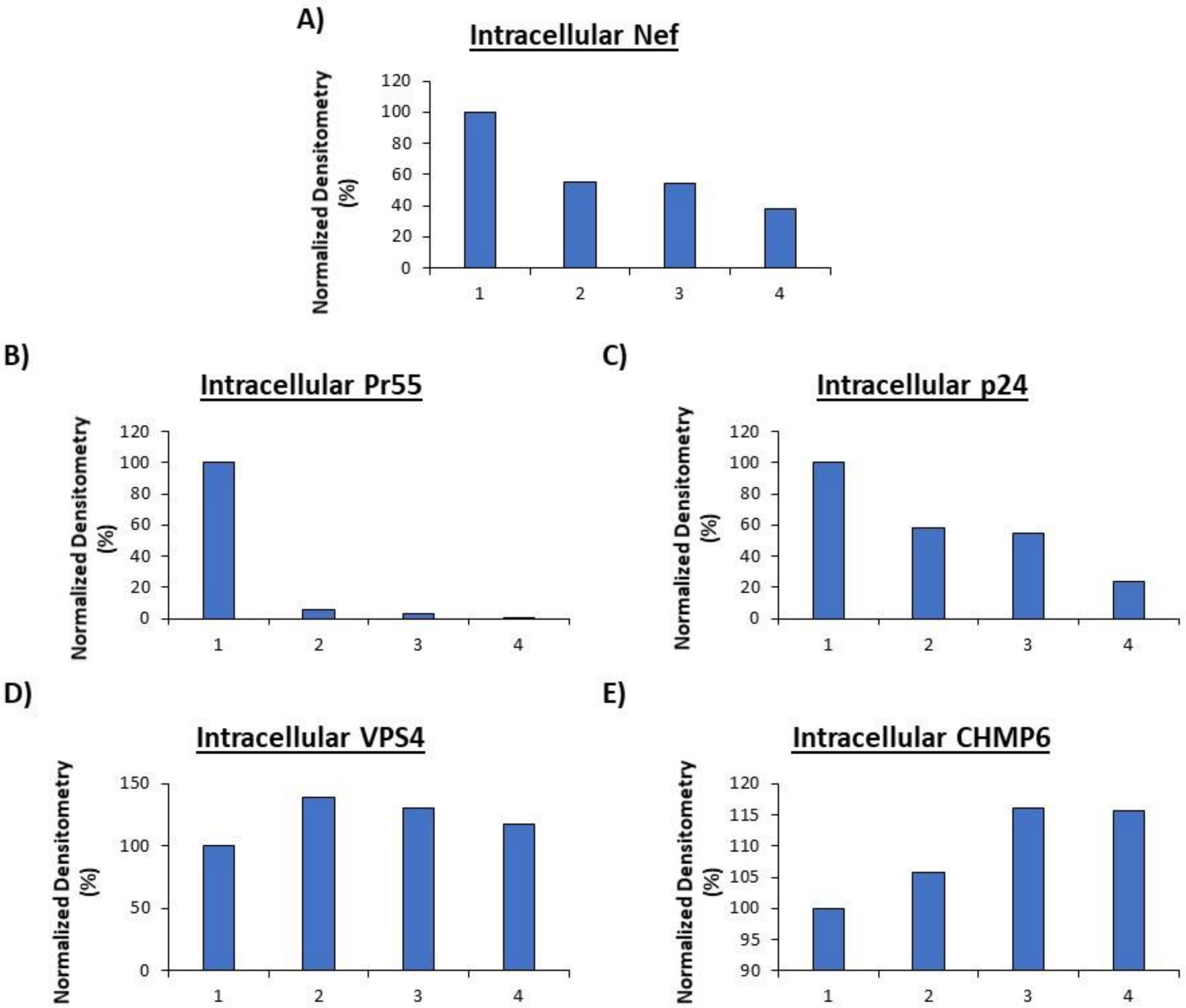

Figure S8. Normalized densitometry analysis of CBD lowering intracellular viral cargo in HIV-1 infected monocytes. (**A**) Nef, (**B**) Pr55, (**C**) p24, (**D**) VPS4, and (**E**) CHMP6 densitometry counts were performed on the Western blot data from Figure 4B, all of which were normalized to Actin levels.

Supplementary Information

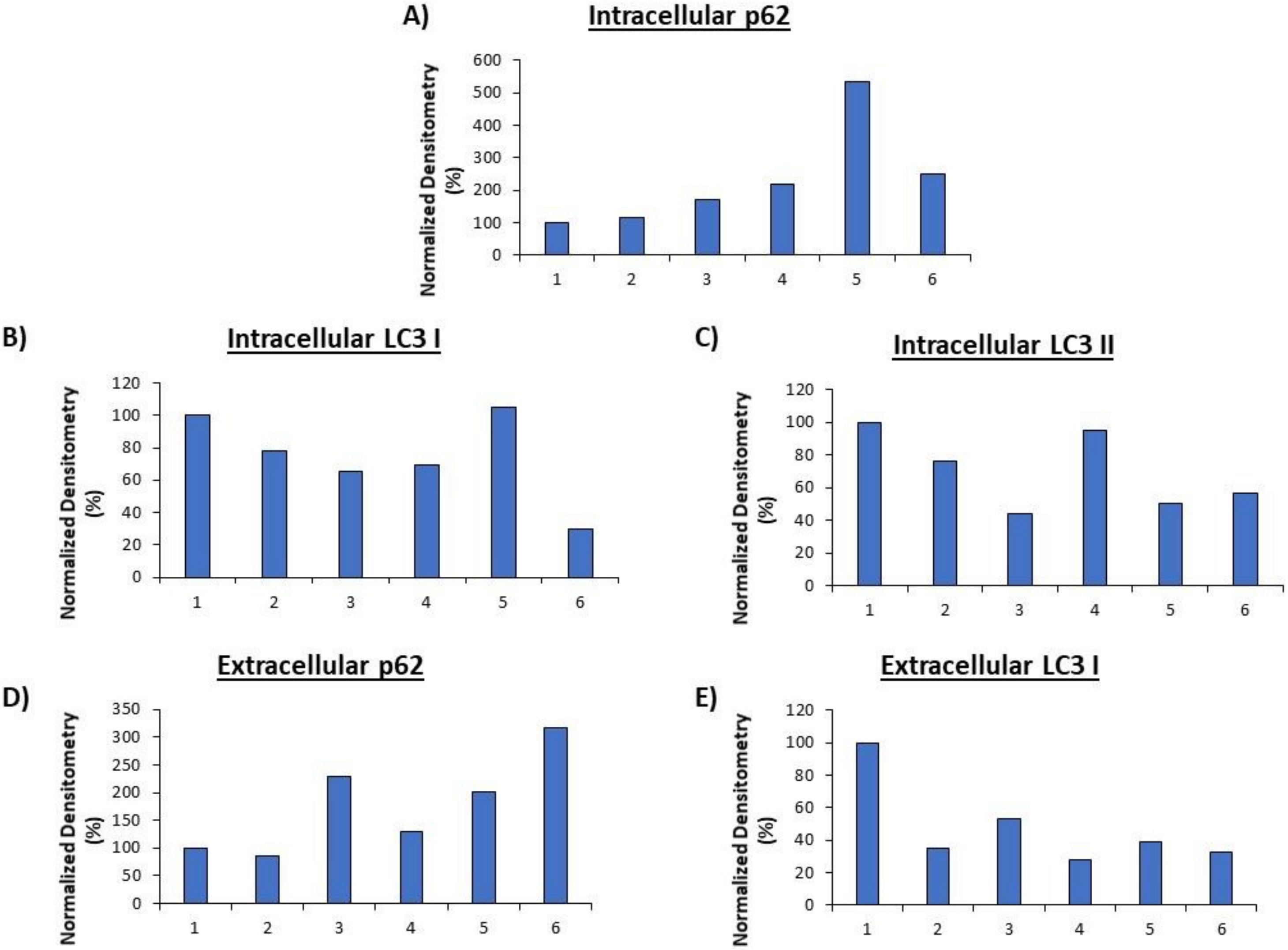

Figure S9. Normalized densitometry analysis of CBD treatment of HIV-1 infected monocytes over time and autophagy. Intracellular (**A**) p62, (**B**) LC3 I, and (**C**) LC3 II levels, and extracellular (**D**) p62, and (**E**) LC3 I levels were counted on the Western blot data from Figure 5A through densitometry analysis, followed by normalization to Actin levels.

Supplementary Information

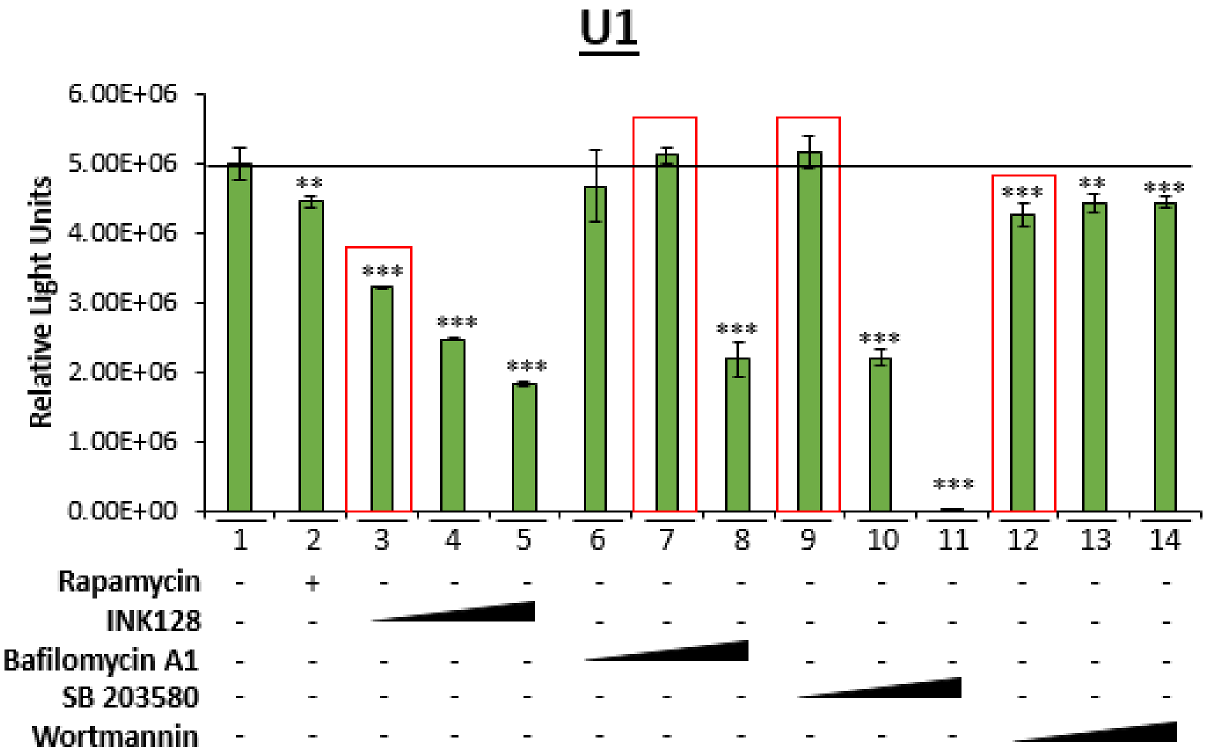

Figure S10. Cell viability analysis of autophagy drugs on HIV-1 infected monocytes and secreted EVs. HIV-1 infected U1 cells (5 x 10^4 cells) were treated with a titration of the following autophagy drugs for 5 days: Rapamycin (50 nM), INK128 (50, 100, and 200 nM), Bafilomycin A1 (10, 50, and 100 nM), SB 203580 (20, 50, and 100 µM), and Wortmannin (2, 10, and 100 nM). Cells were developed and assessed for cell viability using Cell-Titer Glo reagent. Student's *t*-test was used for statistical analysis, where * p-value ≤ 0.05; ** p-value ≤ 0.01, *** p-value ≤ 0.001.

Supplementary Information

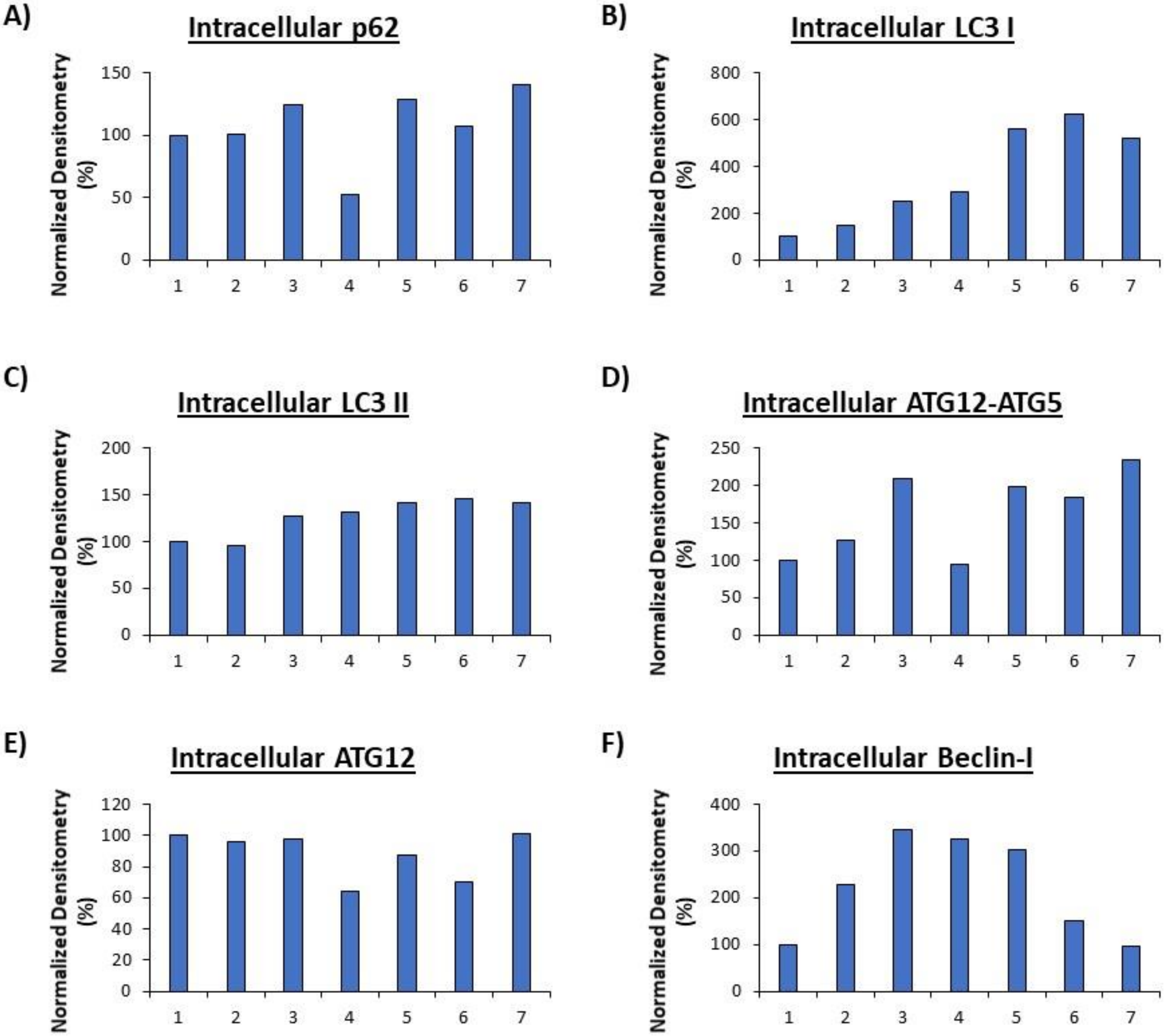

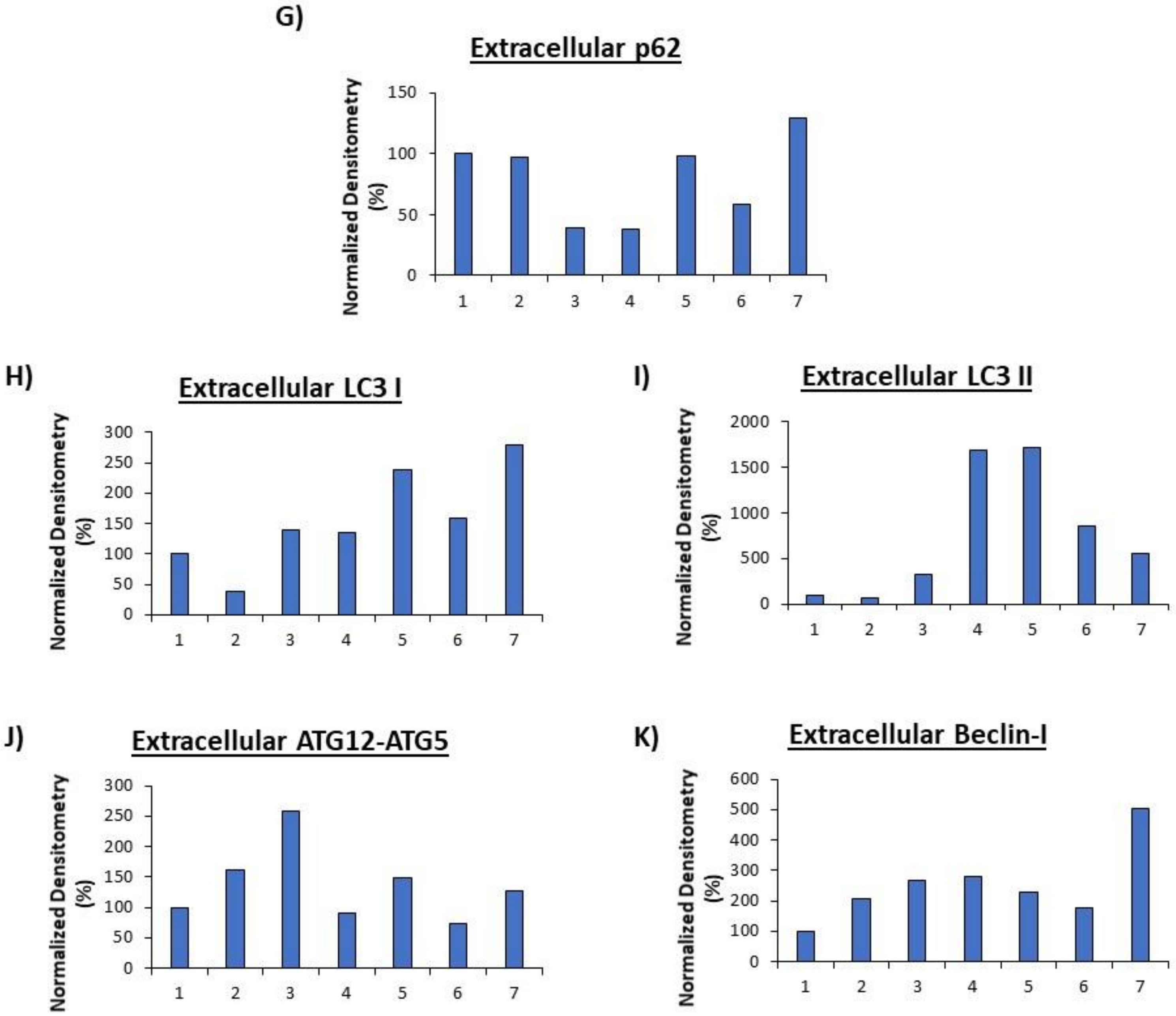

Figure S11. Normalized densitometry analysis of CBD and autophagy drug treatment on HIV-1 infected monocytes. Intracellular (**A**) p62, (**B**) LC3 I, (**C**) LC3 II, (**D**) ATG12-ATG5 complex, (**E**) ATG12, and (**F**) Beclin-1, and extracellular (**G**) p62, (**H**) LC3 I, (**I**) LC3 II, (**J**) ATG12-ATG5 complex, and (**K**) Beclin-1 densitometry counts were calculated from the

Supplementary Information

Western blot data in Figure 5B, normalized to respective intracellular and extracellular Actin levels, and plotted.

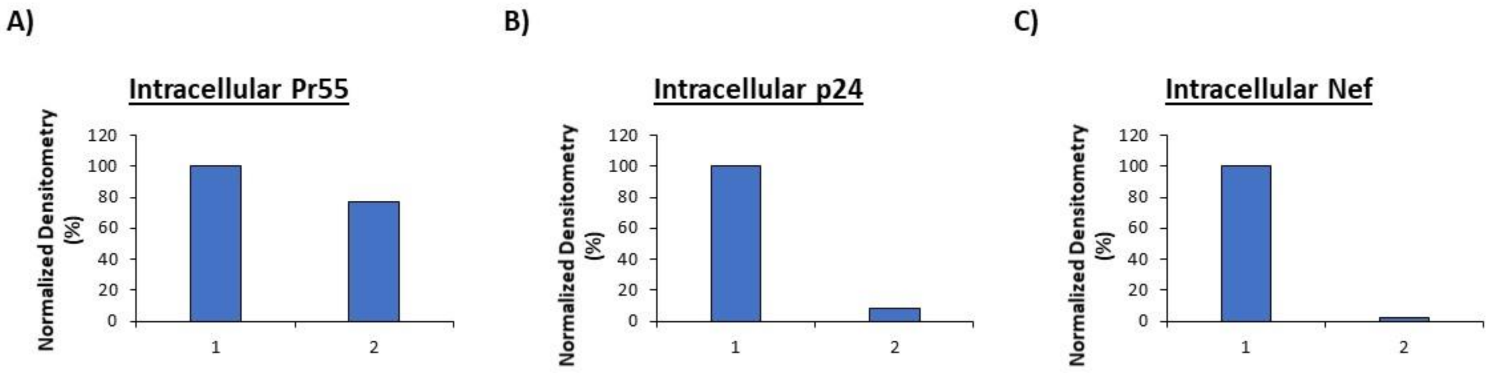

Figure S12. Normalized densitometry analysis of cART on HIV-1 infected 3D neurospheres. Intracellular (**A**) Pr55, (**B**) p24, and (**C**) Nef densitometry counts were analyzed from the Western blot data of Figure 6C, followed by normalization to Actin.

Supplementary Information

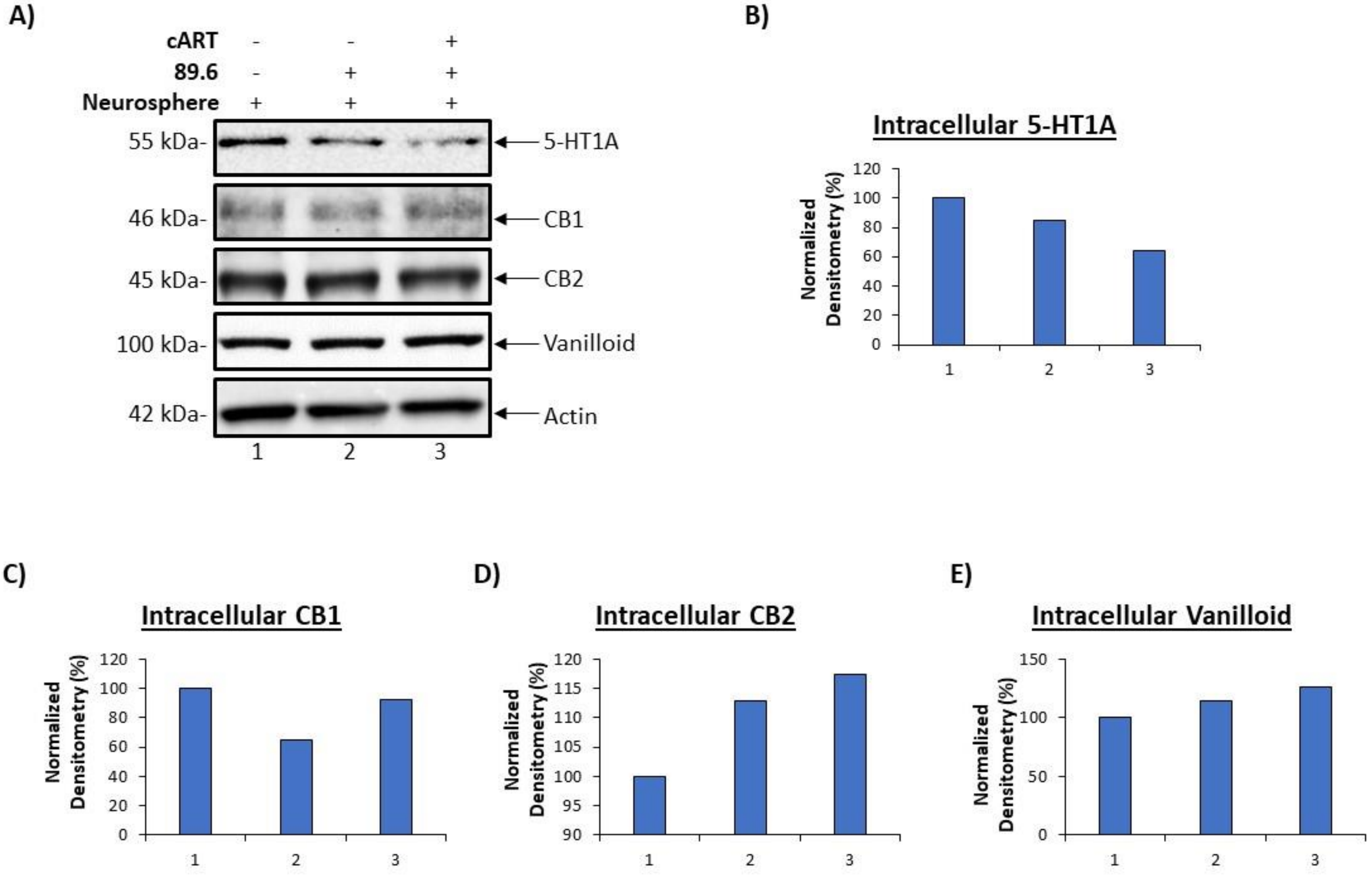

Figure S13. Expression of glial receptors in 3D neurospheres. A) 3D neurospheres were treated ± 89.6 HIV strain ± cART cocktail (10 µM; Tenofovir, Emtricitabine, Lamuvidine, and Indinavir) for a period of 7 days. Neurospheres were harvested, pelleted, and lysed, followed by SDS-PAGE and western blot analysis for neuronal receptor (Vanilloid), cannabinoid receptors (CB1 and CB2), serotonin receptor (5-HT1A), and Actin. Intracellular (**B**) 5-HT1A, (**C**) CB1, (**D**) CB2, and (**E**) Vanilloid densitometry counts were analyzed from panel A, all of which were normalized to Actin.

Supplementary Information

A)

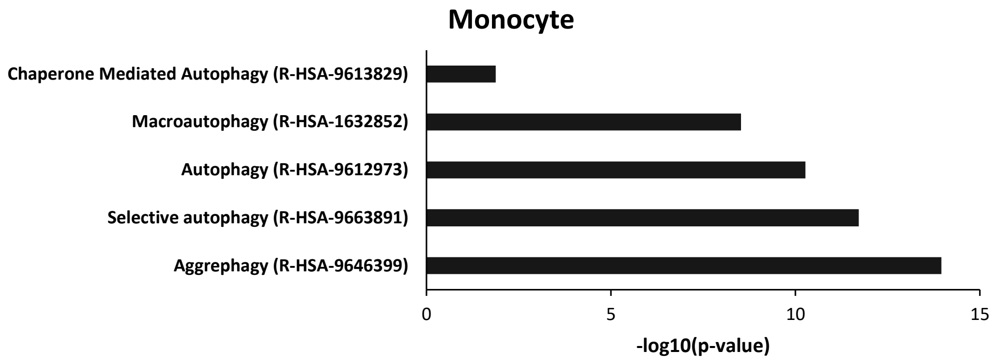

B)

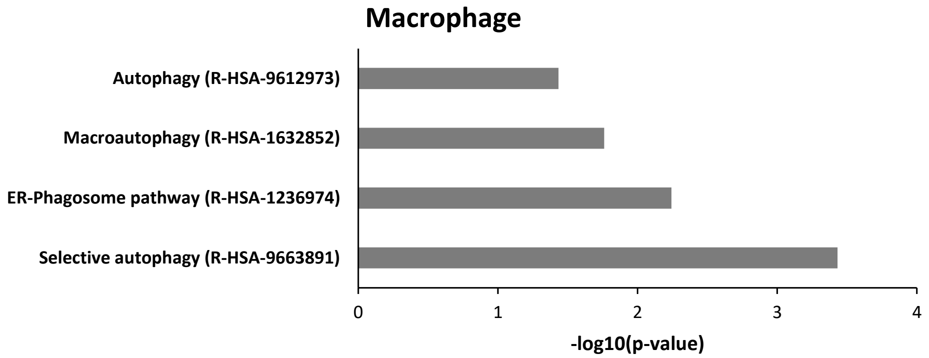

Figure S14. Mass spectrometry analysis of biotinylated CBD pull down from HIV-1 infected lysates. U1 and U1 MDM cells were cultured and lysed. D-Biotin or Biotinylated CBD pull-down from the lysates was performed using Streptavidin-Sepharose beads, which were washed with TNE_{300} (high salt) + 0.1% NP40 buffers. The complexes were analyzed by mass spectrometry. Reactome enrichment analysis was used to assess the CBD pull-down of (**A**) HIV-1 infected monocytes (U1) and (**B**) HIV-1 infected MDM proteins for involvement of the autophagy pathway.
